# Phosphorus-nitrogen systematics of first-generation planetesimals constrain life-essential element delivery to Earth

**DOI:** 10.1126/sciadv.aed8749

**Published:** 2026-06-03

**Authors:** Debjeet Pathak, Rajdeep Dasgupta, Naidhruv Iyer

**Affiliations:** Department of Earth, Environmental and Planetary Sciences, Rice University, MS 126, 6100 Main Street, Houston, TX 77005, USA.

## Abstract

Habitability of rocky planets relies on the budgets of life-essential elements (LEEs) in their building blocks. The provenance and geochemistry of the planetesimals that supplied the LEEs to Earth remain debated. Traditional models argue LEE delivery via outer Solar System chondrites, but their 2 to 4 million-year (Myr) accretion ages preclude them as the first feedstock. To investigate the initial LEE distribution, we reconstructed the phosphorus-nitrogen (P-N) budget of the iron meteorite parent bodies (IMPBs), which accreted <1 Myr of Solar System formation. High-pressure-temperature experiments of P-N partitioning between solid and liquid alloys combined with geochemical models reveal higher P/N ratios in outer Solar System IMPBs than in inner ones—a trend reversed in chondrites. This evolution reflects early refractory schreibersite delivery to the outer disk, later curtailed by Jupiter’s growth. Further modeling in combination with previous elemental and isotopic data on volatile LEEs suggests that both early and later inner Solar System planetesimals are chief contributors to Earth’s LEE inventory.

## Teaser

Distinct P/N of IMPBs to chondrites suggests dual early-late inner Solar System sources of Earth’s life-essential elements.

## INTRODUCTION

Chemical habitability of rocky planetary bodies depends on the availability of life-essential elements [LEEs; i.e., carbon (C), hydrogen (H), nitrogen (N), oxygen (O), phosphorus (P), and sulfur (S)]. The budgets of LEEs in the planets and planetary embryos are chiefly controlled by the LEE budgets in the planetesimals that build them (*1*, *2*). The LEE budget of all putative feedstock planetesimals of Solar System rocky planets, however, remains unconstrained. This is largely because the meteoritic records of early Solar System planetesimals are fragmentary. For example, for the first-generation planetesimals [<1 million years (Myr) from the calcium aluminum inclusions (CAIs)] (*3*), only the crystallized cores are available in the form of iron meteorites. The irons were exposed after the disruption and stripping of the silicate mantle of their parent bodies. Therefore, reconstructing the iron meteorite parent body (IMPB) compositions requires quantitative modeling to undo the differentiation process of core crystallization, metal-silicate differentiation, and any associated atmospheric loss. The compositions of the later formed planetesimals (2 to 4 Myr from the CAIs) (*4*), i.e., the chondrites, cannot be used to model all possible building blocks as the planetesimals are expected to vary temporally and spatially, controlled by the geochemical nature of the stable LEE-bearing phases, which in turn were controlled by the thermal and dynamical evolution of the protoplanetary disk.

Traditional models of LEE delivery to Earth argue for the late contribution of outer Solar System carbonaceous (CC) chondrites to explain the present-day LEE budget of the bulk silicate Earth (BSE) (*5*–*7*). Although evidence in support of this model remains (*8*), studies based on elemental ratios of volatile LEEs, i.e., C/N and C/H, ruled out late accretion of unprocessed chondrites as the primary LEE delivery mechanism to the BSE (*2*). Instead, there is a growing recognition that the delivery of LEEs likely took place throughout the main accretionary phases of Earth (*9*–*12*) with contributions from many generations of planetesimals. If LEE delivery took place throughout the main phases of accretion, the putative planetesimals would likely come not only from the outer Solar System but also from inner Solar System [e.g., (*9*, *13*)], as all models of Solar System formation suggest scattering of outer Solar System chondrites during late phases of terrestrial planet formation [e.g., (*14*, *15*)]. Specifically, some key characteristics of terrestrial volatile LEEs could be contributed by the earliest formed planetesimals in the Solar System, i.e., the IMPBs. However, the feasibility of IMPBs being important contributors to terrestrial LEEs remains uncertain. Like chondrites, iron meteorites also sample different regions of the protoplanetary disk as they are classified into the inner Solar System [noncarbonaceous (NC)] and outer Solar System (CC) on the basis of the stable isotope ratios of refractory elements like molybdenum (Mo) and nickel (Ni) (*3*, *16*, *17*). Therefore, iron meteorites are critical in assessing the possible roles of first-generation planetesimals in bringing LEEs to Earth from different parts of the Solar System.

Most studies on LEE delivery to terrestrial planets thus far focused only on volatile elements, i.e., C, H, N, and S (*1*, *2*, *10**–**12*, *18**–**20*). Delivery of nonvolatile or moderately volatile element P received less attention. The same applies for iron meteorites as well; attempts have been made to reconstruct their volatile elemental budgets, i.e., C, S, and N (*21**–**23*) and isotopes [e.g., (*24*)], but not simultaneously considering the nonvolatile LEEs. Hence, the relative timing of accretion and provenance for volatile versus nonvolatile LEE to terrestrial planets remains unconstrained. Geochemically, N [condensation temperature of <100 K (*25*)] is highly volatile and atmophile in nature (*25*). P, on the other hand, primarily condenses out at as a high-temperature (700 to 1100 K at 10^−4^ atm) (*25*) phase schreibersite [Fe_3_P; (*25*)]. P is mostly non-atmophile. Moreover, P-bearing phases are also stable during low-temperature aqueous alteration processes. This is evident from CC chondrites, which show decreasing P/N with increasing N (fig. S1), reflecting stable P and variable N from parent body alteration. Hence, the ratio of P/N, a nonvolatile LEE to a volatile LEE, needs to be assessed for various putative building block planetesimals for terrestrial planets. While P and N show contrasting behavior during thermal processing and degassing, they both are siderophile under relatively oxidizing conditions [(*1*, *12*, *20*, *26*) for N; (*27**–**29*) for P] and are present as nonmetal light elements in the metallic cores. Although the origin of P on Earth is believed to be via chondritic meteorites (*30*), the late accretion of chondrite cannot explain the P/N ratio of the BSE (fig. S1). The P abundance of the BSE is generally explained by metal-silicate partitioning in a deep magma ocean (MO) (*29*). However, the extreme depletion of the BSE N budget cannot be explained by core formation alone [e.g., (*31*)]. This brings the question of whether the P-N systematics of the BSE required extreme N depletion of the chondritic planetesimals via parent body processes or whether the BSE P-N systematics were rooted to nonchondritic planetesimals having contrasting P-N geochemistry. However, to date, no study has attempted to assess the evolution of P/N across different generations of planetesimals and Solar System regions or linked this to Earth’s LEE budget.

Here, we estimate the P and N inventories of the inner and outer Solar System IMPBs, combining laboratory experiments, constraining P/N fractionation during iron meteorite crystallization, and geochemical modeling. The reconstructed P-N systematics of IMPBs and their comparison with those for chondrites reveal how the nonvolatile to volatile LEEs evolved in the protoplanetary disks from the inner to outer Solar System and from the first generation of planetesimals to the latter. The P/N ratios of the IMPBs and chondrites also underscore the importance of the early inner Solar System planetesimals in LEE delivery to the growing Earth during the main phase of accretion.

### Experimental and analytical procedure

Iron meteorites represent crystallized products of liquid metallic alloys. During crystallization of such alloy melts, elements are redistributed between coexisting solid and liquid phases. Consequently, reconstructing the composition of the parental liquid from which iron meteorites crystallized requires quantifying the partitioning behavior of elements between solid alloy (SA) and liquid alloy (LA). Accordingly, to reconstruct the P and N budgets of IMPBs, it is essential to determine the partitioning of P and N between Fe-rich SA and coexisting LA. Previous studies have used such experimental data and fractional crystallization (*32**–**34*) models of various siderophile element trends in iron meteorites to reconstruct the P budget of the IMPB cores. Similarly, using the experimental constraints (*21*, *22*) in conjunction with the N measurements in iron meteorites (table S3) (*35**–**39*), the N budget of the IMPB cores was reconstructed. However, previous experiments on $D_{N}^{\frac{\text{SA}}{\text{LA}}}$ (N content in SA/N content in LA) were conducted only in the presence of S (*21*, *22*) and C (*22*) and did not consider the effect of P (the second most important nonmetal in IMPB cores after S). Therefore, reliable reconstruction of the P and N budgets of the IMPB cores required mutual partitioning behavior of P and N between the SA and the LA. Therefore, we performed high-pressure-temperature experiments to constrain the simultaneous partitioning of P and N between Fe-rich SA and LA. We conducted the experiments using an end-loaded piston cylinder in crushable magnesium oxide (MgO) capsules with a starting mix of Fe + Ni + P ± S + N at 2 GPa and 1050° to 1600°C (Materials and Methods; table S1). N and other element abundances in SA, LA, and accessory phases were determined using electron probe microanalysis (table S2 and Materials and Methods).

## RESULTS

The experiments produced an Fe-rich SA and an LA. The quenched LA comprises micrometer-sized quench crystals of P-, S-, and Fe-rich domains (fig. S2). A few of our low-temperature experiments also produced troilite and schreibersite, providing partitioning data of N between schreibersite and LA and troilite and LA as well (fig. S2C). The phase assemblage of our experiments as a function of starting composition and temperature aligns with previous studies (*40**–**43*) (Supplementary Materials).

**Fig. 1.**
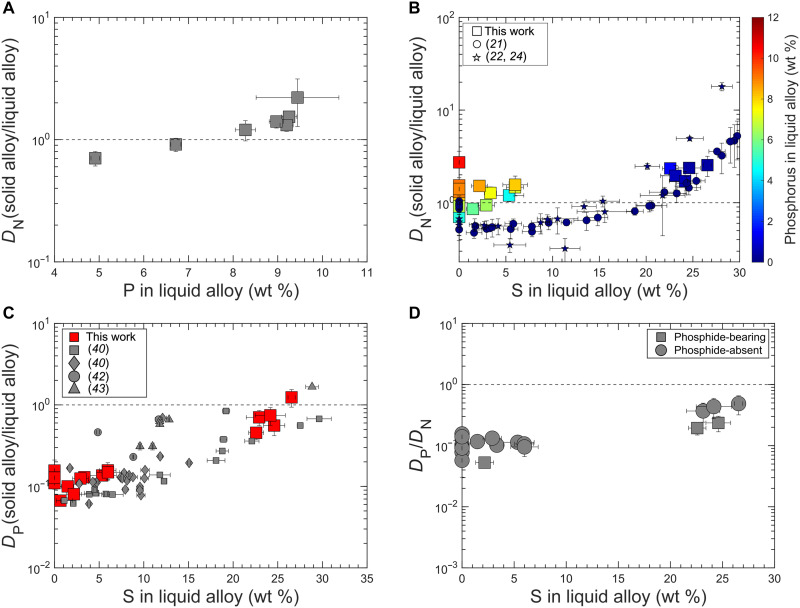
$D_{N,P}^{\frac{\text{SA}}{\text{LA}}}$ as a function of S and P content of the LA. (**A**) $D_{N}^{\frac{\text{SA}}{\text{LA}}}$ for S-free experiments plotted against the LA P content. N becomes mildly compatible in the SA with increasing P content of the LA to ~7 wt %. (**B**) Effect of LA S and P concentrations on $D_{N}^{\frac{\text{SA}}{\text{LA}}}$, which increases with increasing S concentration in the LA similar to the observations in previous studies (*21*, *22*). With low LA S content, a high P concentration in the LA makes N more compatible. However, in high S and low P concentrations, the effect of P is diminished, and $D_{N}^{\frac{\text{SA}}{\text{LA}}}$ is controlled by the LA S content. (**C**) $D_{P}^{\frac{\text{SA}}{\text{LA}}}$ as a function of the S concentration in the LA. $D_{P}^{\frac{\text{SA}}{\text{LA}}}$ increases by an order of magnitude, i.e., from a value of ~0.1 to ~1 with increasing S content of the LA from ~0 to ~25 wt %. In other words, for almost the entire range of S content in the LA of this work and other previous works (grey square, 3 GPa; diamond, 5 GPa) (*40*, *42*, *43*), P remains incompatible. (**D**) $\frac{D_{P}^{\frac{\text{SA}}{\text{LA}}}}{D_{N}^{\frac{\text{SA}}{\text{LA}}}}$ from our experiments against the LA S content. $\frac{D_{P}^{\frac{\text{SA}}{\text{LA}}}}{D_{N}^{\frac{\text{SA}}{\text{LA}}}}$ increases with S content in the LA, yet N remains more SA-loving compared to P for the entire range of LA S content.

Figure 1A shows a plot of $D_{N}^{\frac{\text{SA}}{\text{LA}}}$ against the P concentration in the LA for our S-free experiments. We observe that N is mildly incompatible ($D_{N}^{\frac{\text{SA}}{\text{LA}}}$ of ~0.5 to 1) at low P content (<7 wt %) in the LA to mildly compatible ($D_{N}^{\frac{\text{SA}}{\text{LA}}}$ of ~1 to 2) with P content in the LA increasing from 7 to 9 wt % (Fig. 1A). In Fig. 1B, we plot the $D_{N}^{\frac{\text{SA}}{\text{LA}}}$ for all experiments against their LA S content; the symbols are color coded with the P content of the LA. N is mildly compatible in the SA at ~2 wt % S and high P (~7 to 10 wt %) content in the LA. Similarly, for high S (~22 wt %) and low P (~1 wt %) content in the LA (Fig. 1B), $D_{N}^{\frac{\text{SA}}{\text{LA}}}$ is similar to previous P-free, S-bearing experiments (*21*, *22*). In addition, we observe that $D_{N}^{\frac{\text{SA}}{\text{LA}}}$ decreases with an increase in temperature (fig. S3). We also observe that N is highly incompatible in schreibersite and troilite (fig. S4).

**Fig. 2.**
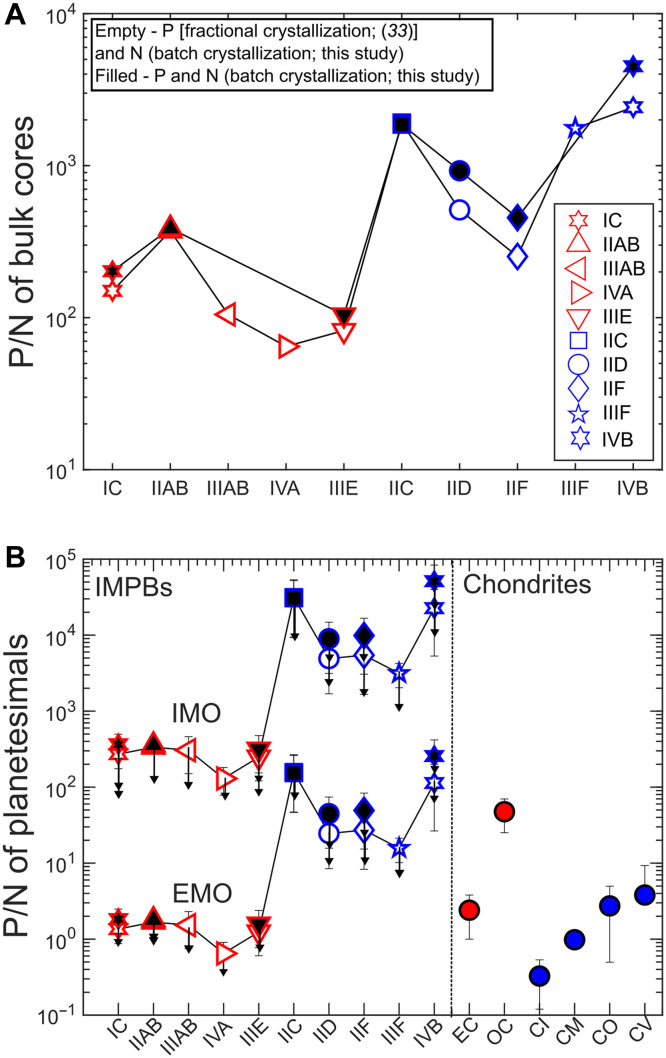
P/N ratio of IMPB bulk cores, IMPBs, and chondrites. (**A**) Estimated P/N (ppm/ppm) of the bulk cores of IMPBs. The empty symbols represent the P estimate from the fractional crystallization model in (*33*) and the N estimate from the batch crystallization model of this study. The filled symbols represent the P and N budgets estimated by batch crystallization models in this study. NC and CC IMPBs are colored red and blue, respectively. NC IMPB bulk cores have relatively lower P/N than the CC IMPBs bulk cores. However, there is overlap between NC group IIAB and CC group IIF. (**B**) P/N (ppm/ppm) of IMPBs estimated for two scenarios of end-member style of differentiation, i.e., IMO and EMO, compared to those for chondrites. The N estimates of IMPBs are from this study, and the P estimates are from this study (batch core crystallization model) and from (*33*) (fractional core crystallization model). The estimated P/N ratios for NC IMPBs are lower than those for CC IMPBs. However, CC chondrites have a lower P/N than the NC chondrites. The downward arrow in the IMPB P/N estimates marks the P/N estimate after considering for N loss during the low-temperature processes leading up to the high-temperature core formation in IMPBs. The N loss is mostly by a factor of ~3 to 5 (fig. S8) (*5*, *47**–**49*, *68*). The estimates for chondrites are from (*5*, *48*, *49*, *68*, *96*). The errors for IMPBs are estimated by propagating the errors in $D_{N,P}^{\frac{\text{SA}}{\text{LA}}},D_{N,P}^{\frac{\text{am}}{\text{sm}}}$, and initial N, P, and S budgets in iron meteorites. Wherever error bars are not visible, they are smaller than the symbols.

In Fig. 1C, we plot the $D_{P}^{\frac{\text{SA}}{\text{LA}}}$ data from experiments against the S concentration in the LA and compare them with previous P-bearing SA-LA experiments (*40*, *42*, *43*). $D_{P}^{\frac{\text{SA}}{\text{LA}}}$ increases with increasing S concentration in the LA, with P becoming mildly compatible at ≥~25 wt % S in the LA. A plot of $D_{P}^{\frac{\text{SA}}{\text{LA}}}$/$D_{N}^{\frac{\text{SA}}{\text{LA}}}$ of our experiments against the S content of the LA reveals that the former increases from ~0.1 to ~1, with the latter increasing from ~0 to 25 wt % (Fig. 1D). Using the new and previous experiments, we parameterized both $D_{N}^{\frac{\text{SA}}{\text{LA}}}$ and $D_{P}^{\frac{\text{SA}}{\text{LA}}}$(figs. S5 and S6; Materials and Methods). Furthermore, we used the batch crystallization model (Materials and Methods) to estimate the N and P budgets of the bulk IMPB cores using a similar approach as used in (*21*). Our batch crystallization model makes use of literature estimates of bulk S, C, and fraction of crystallization of iron meteorites as input parameters (table S3). The P budgets estimated using our batch crystallization model are in agreement with those estimated using the fractional crystallization model (table S5) (*33*). We plot the P/N of the bulk cores in Fig. 2A. Our results show that the cores of NC IMPBs are slightly lower in P/N than those of the CC IMPB cores. However, the P/N of IIAB and that of IIF overlap (Fig. 2A). This is primarily due to the higher P budget of the bulk core of IIAB than the other NC IMPB cores (*34*, *35*)*.*

## DISCUSSION

### P/N of the IMPBs constrains the evolution of the protoplanetary disk

The observed differences in P and N budgets between the bulk NC and CC IMPB cores suggest that inventories of these elements in the earliest formed Solar System planetesimals could have been different as a function of distance from the proto-Sun. If correct, this highlights the potential of the P/N ratio as a powerful tool for probing chemical gradients and processing in the early protoplanetary disk. However, the first one needs to assess whether the modeled variation in the bulk cores propagates to a difference in the estimate of the P and N budgets of the IMPBs. To that effect, first, we estimate the P and N budgets of the silicate reservoir using $D_{N,P}^{\text{alloy melt (am)/silicate melt (sm)}}$ [for N, (*44*); for P, (*29*)] and our bulk core estimates (table S5), taking into consideration the IMPB’s redox state during differentiation (*45*). The appropriate $D_{N,P}^{\frac{\text{am}}{\text{sm}}}$ for the differentiation of different IMPBs is documented in table S4 and in fig. S7. In fig. S7, we observe that $D_{N}^{\frac{\text{am}}{\text{sm}}}$ is greater than $D_{P}^{\frac{\text{am}}{\text{sm}}}$ for all of the IMPB groups with the exception of group IIAB as IIAB is substantially more reduced than the other NC IMPBs and CC IMPBs. Subsequently, we estimate the P and N budgets of the bulk IMPB using our estimated values and the core mass fraction (*46*) and the oxygen fugacity (*f*O_2_) of the IMPBs (*45*).

To estimate the P/N ratio of the IMPBs and propagate uncertainties, we consider two contrasting differentiation regimes (Fig. 2B)—internal MO (IMO) (*21*, *23*, *47*) and external MO (EMO) (*21*, *23*, *47*) via which IMPBs might have differentiated (Materials and Methods). In IMO, the P/N ratio reconstruction is affected by distribution of these elements between the core and the mantle, and in EMO, the reconstruction involves contributions from the core, mantle, and atmosphere. Along with the IMPBs, we plot the P/N ratio of inner Solar System enstatite and ordinary chondrites and outer Solar System CC chondrites (Fig. 2B). We observe a systematic gradation in P/N ratios from the inner to outer Solar System IMPBs, irrespective of whether they underwent the IMO or EMO style of differentiation. NC IMPBs exhibits lower P/N than the CC IMPBs.

Iron meteorite groups that previously appeared to overlap in P/N, on the basis of their bulk core composition, such as the NC groups IIAB and IIF, show a clear separation in P/N of the entire parent bodies. This difference arises because of the distinct *f*O_2_ conditions prevailing during core formation. Group IIAB, which formed under highly reducing conditions [IW-3.2, where IW refers to log*f*O_2_ value equivalent to that set by equilibrium between iron (Fe) and wüstite (FeO); (*45*)], retains very little P in the silicate fraction, as the majority gets partitioned into the metallic core because of the strongly siderophile behavior of P at low *f*O_2_ (*29*). In contrast, group IIF formed under comparatively oxidizing conditions [IW-1.2; (*45*)], allowing a larger fraction of P to remain in the silicate portion. Consequently, reconstructed bulk P/N ratios are higher for IIF, which receives greater contribution from the silicate reservoir, than for IIAB, despite the compositional similarity between the two iron groups in terms of their bulk cores’ P/N signature (Fig. 2A).

The P/N of IMPBs for the IMO scenario plots higher than all chondrites (Fig. 2B). However, in the case of the EMO scenario, there is an overlap of a few NC and CC IMPBs with the estimates of chondrites, as significantly more N is estimated for the IMPBs (Fig. 2B). Notably, the CC IMPBs have a higher P/N than the NC IMPBs (Fig. 2B). The N budget estimated by our method accounts only for the N that was available in the IMPBs at the onset of core formation. There could be loss of volatile LEE such as N during heating of IMPBs, as evidenced from chondrites, where bulk N abundances (*5*, *48*, *49*) decrease mostly by a factor of ~3 to 5 and do not exceed a factor of ~10 with increasing effective metamorphic temperature (fig. S8) (*50*). If IMPBs experienced a similar extent of N loss during heating, the difference in P/N ratio between the NC and CC IMPBs still exists even after accounting for the loss (Fig. 2B). Even if only the CC IMPBs experienced pre–core formation N loss, the CC IMPBs still exhibit higher P/N ratios than the NC IMPBs (Fig. 2B). However, for the later forming chondritic planetesimals, the trend gets erased and reversed, with inner Solar System enstatite and ordinary chondrites having a slightly higher P/N than the outer Solar System CC chondrites (Fig. 2B). The observed difference in P/N ratios between the first-generation planetesimals and the later forming planetesimals suggests a temporal evolution in the P/N signature of the protoplanetary disk.

**Fig. 3.**
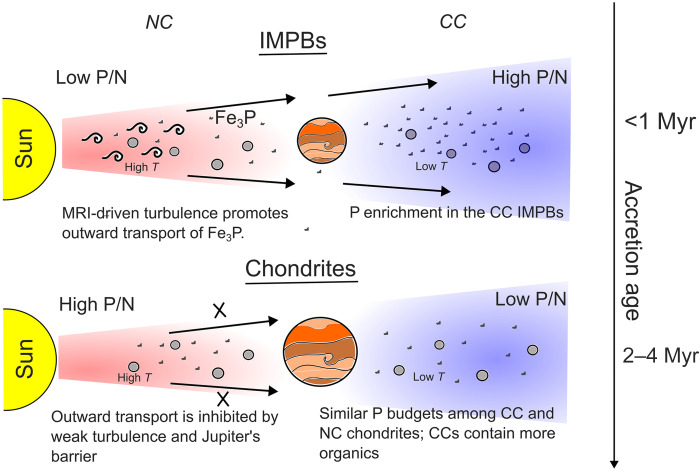
Cartoon showing the protoplanetary disk evolution explaining the P-N systematics of IMPB and chondritic planetesimals. In the early stages of the protoplanetary disk, strong turbulence driven by MRI and higher temperature resulted in outward flow of refractory material along the midplane of the protoplanetary disk toward the outer Solar System region. The migration resulted in the enrichment of P (P-bearing schreibersite denoted as small grains in the figure) in the outer Solar System CC IMPBs. However, because of the weakening of the outward flow and blockage by Jupiter in the later stages of the protoplanetary disk, the P budget became similar in the inner and outer Solar System. Conversely, the N budget gradient observed where NC IMPBs grade into higher budgets than the CC IMPBs can be explained by the higher stability of refractory N-bearing material in the reduced inner NC region than in the CC region where oxidation-induced breakdown of N-bearing refractories might be common. Moreover, the breakdown of N-bearing organics in the CC IMPBs at core-forming temperatures might result in a lower budget of N in CC IMPBs than in the refractory, N-enriched NC IMPBs.

The P/N signature of IMPBs is controlled by the geochemical nature of the accreting phases and the parent body processes. The availability and abundance of these accreting phases are regulated by the combined effect of the dynamics and the temperature structure prevailing in the disk. P primarily crystallizes as the refractory phase schreibersite (*25*, *51*) from the protosolar nebula in the hotter inner Solar System regions (Fig. 3). We argue that the turbulent inner solar disk (Fig. 3), driven by magnetorotational instability (MRI) (*52**–**54*) under elevated temperatures, produced strong midplane outflows (*55*, *56*) that transported significant proportion of refractory schreibersite from the hot inner disk (*57*) to the cooler outer disk (Fig. 3). These strong outflows (*54*, *56*, *57*) also transported massive amounts of CAIs to the accretion zone of CC IMPBS (*33*) and CC chondrites (*58*). In contrast to the P budget, the N gradation of the IMPB cores (table S5 and Fig. 2A) has a minimal contribution to the observed P/N signature (Fig. 2A), albeit in the same direction as the effect of P. If N-bearing refractories (TiN and CrN) (*59*) traveled similarly alongside schreibersite and CAIs during the midplane outflows, they might have undergone oxidation in the outer Solar System regions, resulting in the volatilization and depletion of N. A combination of these processes contributed to the N budget gradation observed between NC and CC IMPB cores (table S5).

The variation in P/N among CC and NC IMPBs disappears and reverses in the later-generation planetesimals, i.e., chondrites. CC chondrites have P/N ratios even lower than the enstatite and ordinary chondrites (Fig. 2B). The major cause is the reduced outflow of material primarily due to the cessation of the MRI-driven turbulence that operated during the high-temperature earliest Solar System periods (*60*, *61*). The blockage of refractory material transport by Jupiter (*3*, *9*) or pressure bumps (*62*) could also result in nearly equalizing the P budget in both the inner and outer Solar System regions. The solar disk also cooled further in 3 to 4 Myr to help stabilize N-bearing organics in the outer disk, lowering the P/N ratio of CC-chondritic planetesimals.

### NC IMPBs as a source of Earth’s LEE budget

The accretion of terrestrial planets started early during the protoplanetary gas disk phase. The oligarchic growth model of terrestrial planets argues for the involvement of planetesimals and planetary embryos that were already formed by then. On the basis of the isotopic systematics of refractory elements, the main phase of Earth’s accretion [~92% of Earth’s mass (*63*, *64*)] was dominated by enstatite chondrite-like material. However, the enstatite chondrite model [~0.75 for enstatite high iron (EH) and ~0.85 for enstatite low iron (EL) (*65*)] cannot explain the Mg/Si ratio of Earth’s mantle [~1.25 (*65*); ~1.21 (*65*, *66*)], hinting at the likely contribution of other planetesimals, beyond enstatite chondrites, during the early stages of Earth’s accretion. Outer Solar System chondritic planetesimals might have contributed, but isotopic systematics of Mo, Ti, and Cr (*67*) limit their input to Earth to <4%. Therefore, constraining the possible role of the other planetesimals, beyond the chondrites, such as the IMPBs, is critical for understanding the present-day BSE’s LEE inventory. To evaluate such a possibility, we performed a multistage accretion model to determine the P/N composition of the BSE. For our model, we use the IMPB bulk P and N estimates for the IMO style of differentiation as they more realistically represent the geochemical nature of the differentiated planetesimals before their accretion on Earth. Planetesimals that have undergone the IMO style of differentiation represent the silicate and metallic reservoirs, which would be the case for IMPB-sized planetesimals, as the degassed atmosphere would be completely lost because of their weak gravitational force (*47*). We performed our calculations for three different initial P/N ratios (table S6) and three different redox evolutionary pathways (fig. S9A). The three different initial P/N ratios correspond to P and N budgets as estimated in this study for NC IMPB and CC IMPB and as measured in enstatite chondrites (*68*). Although the IMPBs and even some of the chondrites are expected to be differentiated at the time of proto-Earth’s accretion, we treat the bulk P and N inventories of core + silicates as the initial concentrations that undergo alloy-silicate fractionation on growing Earth. This is because of the fact that the bulk scale core merger and only the silicate portion of the differentiated body contributing to the BSE are possibly relevant only for giant impacts. In case of all other small bodies such as planetesimals, accretion has been shown to take place via complete dispersal of the metals, forming small droplets sinking to form Earth’s core [e.g., (*69**–**72*)]. In such scenarios, the P and N budgets of both the cores and silicate reservoirs of planetesimals take part in setting the BSE inventories through repartitioning in growing prototerrestrial MO.

**Fig. 4.**
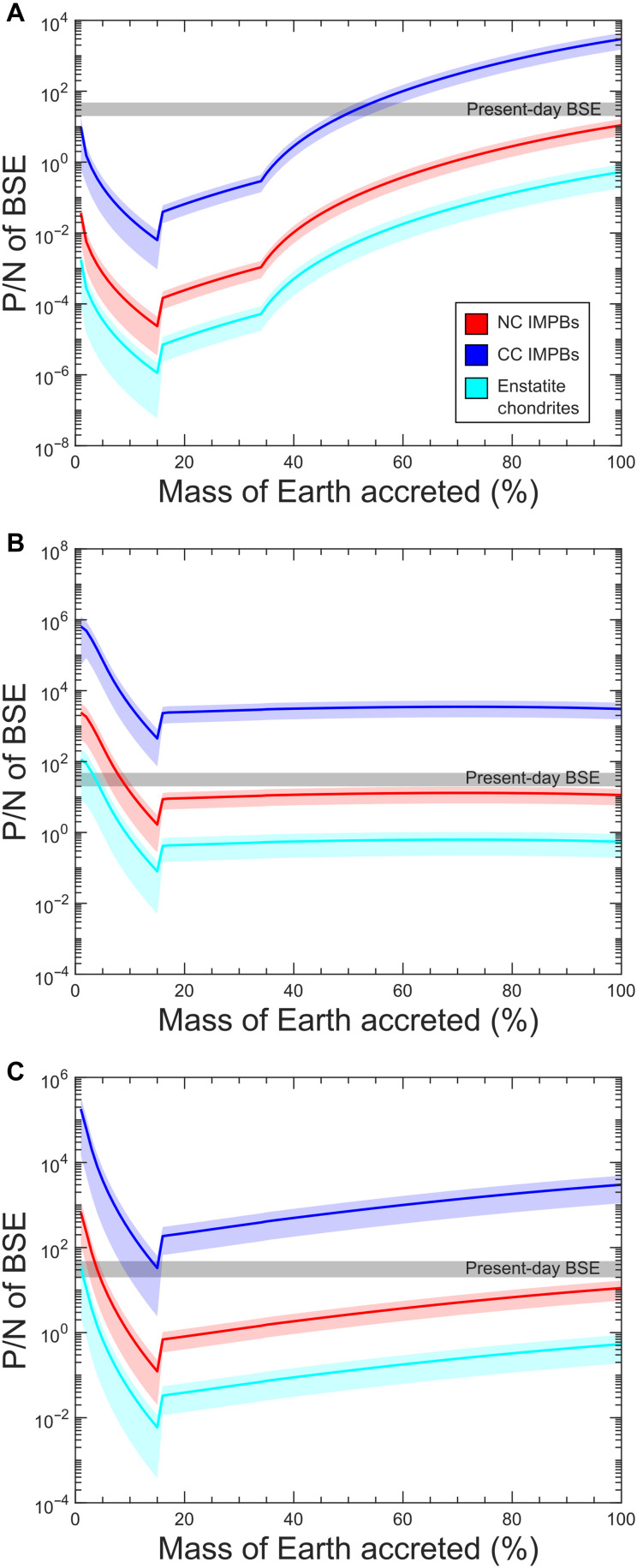
P/N in the silicate reservoir of model Earth as a function of mass accreted for different planetesimal building blocks. The gray band represents the present-day BSE estimate (*66**,* *97*). The shaded region along the curves represents the uncertainty in the modeled values, which is estimated by propagating the errors in the estimated $D_{N,P}^{\frac{\text{am}}{\text{sm}}}$. The calculation was performed for three redox evolution pathways: (**A**) reduced: reduced material to more oxidizing material; (**B**) oxidized: oxidizing material to more reduced material; (**C**) homogeneous: accretion of material having similar redox characteristics throughout accretion. Irrespective of the redox pathway, CC IMPBs produce P/N greater than the present-day BSE. Enstatite chondrites generate P/N below the present-day BSE’s value by at least one order of magnitude. However, the NC IMPBs produced P/N below the present-day BSE by only a factor of 2, suggesting that only a modest N loss during accretion involving NC IMPB materials is necessary to satisfy the P-N characteristics of the BSE.

The model results are dependent on the partitioning of P and N between the alloy core and the silicate mantle, which itself is controlled by the pressure, temperature, and *f*O_2_ conditions prevailing during core formation. Under reducing conditions, P prefers alloy melt over silicate melt (*29*, *73*). However, N (*1*, *10*, *18*, *20*, *44*), in reduced conditions, prefers silicate melt over alloy melt (fig. S9B). Our model result (Fig. 4A: reduced; Fig. 4B: oxidized; Fig. 4C: homogeneous) clearly shows that accretion of bodies having an initial P/N signature of enstatite chondrites (Fig 4; depleted by two or three orders of magnitude from the BSE) cannot match the BSE’s P/N signature irrespective of the redox pathway undertaken during accretion. Likewise, accreting only CC IMPBs yields P/N ratios higher (by at least an order of magnitude) than the BSE (Fig. 4), which is problematic given the absence of a viable mechanism to lower the P/N ratio postaccretion. In contrast, regardless of the redox evolution during accretion, accreting only NC IMPBs yields P/N ratios that are slightly below (Fig. 4; by a factor of ~2) the present-day P/N signature of the BSE. This interpretation is consistent with work in (*74*) indicating that Earth formed largely from inner Solar System material, with some compositional differences relative to chondrites. To further assess model robustness, we track the absolute N budget of the silicate reservoir as a function of the fraction of Earth’s mass accreted. We find that no single end-member, i.e., NC IMPBs, CC IMPBs, or enstatite chondrites, perfectly reproduces the present-day BSE N inventory across all redox pathways (fig. S10). However, NC IMPBs provide the closest match: For all redox scenarios, the modeled BSE N abundance is depleted by only a factor of ~3.5 relative to current estimates (fig. S10). This contrasts with the CC IMPB case, in which N is depleted by at least an order of magnitude, and the enstatite chondrite scenario, which yields the BSE N abundances enriched by less than an order of magnitude.

### Ruling out chondritic delivery and N degassing as the chief causes in setting the BSE LEE inventories

For accreted planetesimals with sub-BSE P/N (NC IMPBs and enstatite chondritic planetesimals) or higher N than the BSE (enstatite chondrites), processes such as N loss during degassing can elevate the P/N to match the BSE levels. Such N loss could not only take place during the parent body processes in the planetesimals, which we showed was not sufficient, but also during Earth’s growth and MO degassing during giant impacts. Therefore, N abundance or P/N ratio alone cannot rule out the feasibility of chondrites serving Earth’s sole building blocks. However, for planets ranging in size from Mars to Earth, N is mostly sequestered in the cores (*1*, *75*), resulting in a smaller budget of N in the silicate reservoir available for degassing. Moreover, degassing during MO evolution would not be restricted to N alone; other LEEs, particularly C and H, would also be lost (*75**–**77*). Experimental and modeling studies indicate that under reducing, core-forming MO conditions, C is primarily degassed as CO and is degassed more efficiently than N (*77**–**80*). If such degassed C and N were subsequently lost from Earth’s atmosphere, this process would not lead to the observed superchondritic C/N ratio in the BSE. Consequently, although MO degassing could reduce the N budget and potentially account for the observed P/N ratio of the BSE, it cannot simultaneously reproduce other LEE ratios, particularly those involving C.

Moreover, N delivery solely via CI-chondrites followed by degassing from the MO is also problematic to explain the N isotopic signature of the present-day BSE. CI-chondrite exhibits positive δ^15^N [= (^15^N/^14^N)_sample_/(^15^N/^14^N)_standard_ − 1) × 1000] values, with an average of ~+44‰ (*5*, *49*), whereas the analysis of mid-ocean ridge basalts suggests that the BSE is −5‰ (*81*). Any kinetic degassing of N from the MO would further enrich the residual silicate reservoir in ^15^N, driving δ^15^N to even higher values and resulting in the mismatch with the observed BSE composition. In contrast, enstatite chondrites and NC IMPBs (*9*, *59*), which have N isotopic compositions closer to that of the BSE and primordial mantle [<−40‰, based on the N isotopic composition of peridotitic diamonds; (*81*)], represent more plausible sources for reproducing the present-day BSE’s N isotopic signature. These arguments are also consistent with the previous observation that the BSE N-isotope value is between the CC and NC iron meteorites, with NC irons displaying negative δ^15^N values and falling closer to the BSE (*9*). Therefore, to account for the P/N of the BSE without degassing of N would require the building blocks to have N budget somewhere near the estimates of the NC IMPBs and enstatite chondrites. However, while our accretion model explores end-member scenarios, a more realistic case likely involved contributions from both generations of inner Solar System planetesimals, i.e., enstatite chondrites and NC IMPBs.

In summary, our experimental data and associated geochemical modeling on the reconstruction of the P-N systematics of the first generation of Solar System planetesimals suggest that such characteristics were different from the later-generation planetesimals, i.e., chondrites. The P/N gradient from the inner to outer disk reversed through time, within the first 2 to 4 Myr. Such evolution of the P/N signature indicates the efficient outward flow of refractory schreibersite during the formation timescale of first-generation planetesimals; however, such outflow was later restricted in the disk owing to dissipated outflow, the growth of Jupiter, or the development of pressure bumps. N-bearing organic enrichment in the outer disk was also favored during chondrite formation times, owing to the gradual cooling of the protoplanetary disk. The result is a distinct budget of volatile to nonvolatile LEEs between the first-generation and later-generation Solar System planetesimals. Forward models of the P/N ratio and the N budget of Earth’s silicate reservoir further suggest that first-generation planetesimals from the inner Solar System are viable candidates to bring both P and N to Earth, whereas outer Solar System reservoirs fail to satisfy the P/N ratio of the BSE. While N loss via MO degassing can elevate the P/N ratio, such a process fails to explain the superchondritic C/N of the BSE and also causes further mismatch of the N-isotope composition of the BSE. Our study obviates the need for volatile-rich material delivery and subsequent volatile loss to explain the BSE LEE inventories. We instead show that both volatile and nonvolatile LEEs can be sourced from the inner Solar System. Proper characterization of other elements in iron meteorites and reconstructing the elemental abundance of elements in the stripped silicate reservoir of NC IMPBs would provide more refined insight into the possible role the NC IMPBs played in shaping the geochemical nature of Earth.

## MATERIALS AND METHODS

### Starting compositions

The starting composition was composed of a homogeneous mixture of reagent-grade powders of Fe, Fe_3_P, FeS, Fe_3.3_N, and Ni powder. The powders were weighed using a microbalance, mixed using an agate mortar under ethanol, and dried overnight. The dried powders were stored for more than 72 hours in a desiccator. Several starting mixes were prepared, keeping the bulk N concentration fixed at 0.5 wt % but with the S and P contents of 0 to 15 wt % and 1 to 12 wt %, respectively. The Ni content was varied between 0 and 8 wt %.

### Experimental procedure

The experiments were performed in an end-loaded piston cylinder apparatus; ^1^/_2_-inch (1.27 cm) BaCO_3_ assemblies, with crushable MgO spacers; and straight-walled graphite furnaces at Rice University, Houston, TX. The *P*-*T* calibration of the experimental assembly was after (*82*). The experiments were performed at a fixed pressure of 2 GPa. It is estimated that the pressure in the center of the IMPBs is ~1 GPa (*83*). Moreover, small protoplanets ranging in the size of Vesta can have pressures in the centers up to around 1 to 2 GPa (*83*). Therefore, our choice of 2 GPa, although higher than the estimated pressure range, would still be within the pressure range of protoplanets. Moreover, previous experiments have shown that pressure does not significantly affect the partitioning of elements between SA and LA (*21*, *84*). The pressure choice of 2 GPa was also prompted by the technical challenge of avoiding thermocouple oxidation at lower-pressure, high-temperature experiments using a piston cylinder. The experiments were performed in crushable MgO capsules. All experiments were sintered at 750° to 800°C at the target pressure for 8 to 12 hours to reduce the porosity of the MgO capsules. Following the sintering step, the experiments were heated to the target temperature at a rate of 100°C/min and held for 1 to 40 hours. The experimental temperatures were monitored and controlled using a type-C thermocouple, and the experiments were terminated by cutting off power to the heater. Following depressurization, the samples were recovered and mounted longitudinally in Petropoxy 154 and polished using SiC sandpapers down to 1200 grit size. Last, the samples were polished on a velvet cloth using alumina slurry down to 0.3 μm. Water was used as lubricant for polishing.

### Analytical procedure

The polished samples were carbon coated and studied using a JEOL JXA8530F Hyperprobe EPMA equipped with five wavelength-dispersive spectrometers at the Department of Earth, Environmental and Planetary Sciences at Rice University. The beam conditions were a 15-kV accelerating voltage and 50-nA beam current. A beam diameter of 1 μm was used for troilite and phosphide phases. The Fe-rich SA and LA were measured using 20-μm and 5- to 20-μm beam diameters, respectively. Counting times for N were 80 s on the peak and 60 s on each background. For the rest of the elements (Fe, Ni, S, P, and O), counting times of 10 s on the peak and 5 s on the lower and upper backgrounds were used. Detection limits of ~170 and ~49 parts per million (ppm) were achieved for N and P, respectively, in troilite; ~12 and ~190 ppm for P and N, respectively, in schreibersite; and ~250 and ~50 ppm for N and P, respectively, in both SA and LA.

### Parameterization of $D_{N}^{\text{SA}/\text{LA}}$ and $D_{P}^{\text{SA}/\text{LA}}$ as a function of LA composition

To use our experimental data of $D_{N}^{\text{SA}/\text{LA}}$ and $D_{P}^{\text{SA}/\text{LA}}$ for estimating bulk core compositions of different IMPBs, we developed parametric equations. For N, first, we use regression models to fit our experimental data to establish a parametric equation for calculating $D_{N}^{\text{SA}/\text{LA}}$ for P-bearing, S-free LA. Following the model in (*85*), the relevant equations are as follows

$$\text{ln}\left(\frac{1}{D_{N}^{\frac{\text{SA}}{\text{LA}}}}\right)=\beta_{P}\text{ln}\left(\text{Fe domains}\right)-\text{ln}\left(D_{0}\right)$$ (1)

$$\text{Fe domains}=\frac{\left(1-4X_{P}\right)}{\left(1-3X_{P}\right)}$$ (2)

where $X_{P}$ is the mole fraction of P in the LA. The values of the coefficients with 95% confidence intervals (in parentheses) are as follows: β_P_ = 3.31 (−0.54, 6.06), $\text{ln}\left(D_{0}\right)$ = 0.61 (−0.12, 1.35). The regression fit is presented in fig. S5.

The resultant $D_{N}^{\text{SA}/\text{LA}}$ by considering both P and S in the LA can be written similarly as follows

$$\text{Fe domains}=\frac{\left(1-2X_{S}-4X_{P}\right)}{\left(1-X_{S}-3X_{P}\right)}$$ (3)

$$\beta_{P+S}=\left(\frac{2X_{S}}{2X_{S}+4X_{P}}\right)\beta_{S}+\left(\frac{4X_{P}}{2X_{S}+4X_{P}}\right)\beta_{P}$$ (4)

where $X_{S}$ the mole fraction of S in the LA, and β_S_ is adopted from (*21*).

For estimating the P content in the equilibrium LA, we use the $D_{P}^{\text{SA}/\text{LA}}$ calculated from the parameterization of P partition coefficient between the SA and the LA. The parameterization that we use to fit our data and previous data (*40*, *42*, *43*) is based on the equation prescribed in (*40*)

$$\text{ln}\left(D_{P}^{\frac{\text{SA}}{\text{LA}}}\right)=a+b\text{ln}\left(X_{S}\right)$$ (5)

where $X_{S}$ is the mole fraction of S in the LA. The coefficients (with 95% confidence intervals in parentheses) are as follows: *a* = −5.21 (−6.36, −4.07), *b* = −2.5 (−2.80, −2.30). The regression fit is presented in fig. S6.

### Estimating the N and P budget of the IMPBs

To estimate the N and P contents of the IMPBs, first, we need to estimate the N and P of the bulk cores of IMPBs. Once the bulk molten core compositions are determined, one can reconstruct the N and P of the IMPBs by calculating the equilibrium content of these elements in the unsampled reservoirs of the IMPBs, i.e., molten silicate ± atmosphere.

To estimate the N and P content of IMPB bulk cores, we used the measured N and P abundance (table S3) of iron meteorites along with the parameterized experimental partition coefficients of both N and P in metallic systems, i.e., $D_{N,P}^{\frac{\text{SA}}{\text{LA}}}$ (Eqs. 1 and 5). Previous studies (*21*, *22*) have only looked at the effect of S in the LA to estimate $D_{N}^{\text{SA}/\text{LA}}$. In this study, we use our new parameterization that captures the combined effect of both S and P in the LA to estimate $D_{N}^{\text{SA}/\text{LA}}$. For P, we use the new parameterization discussed in the previous section. We use a modeling methodology similar to (*21*) to estimate the N content of the IMPB bulk cores. The lack of N versus other nonvolatile siderophile element trend prevents using fractional crystallization models to estimate the N budget of the IMPB cores (*21*, *22*). Therefore, to use the entire range of possible N and P concentrations in iron meteorites and their extent of crystallization (deduced from the trends of nonvolatile siderophile element like Au-As, Ge-As, etc.), we use a Monte-Carlo–based sampling method to randomly select the input parameters (concentrations of N and P in the crystallized SA and fraction of the core crystallized) 10,000 times and use them in the batch crystallization equation

$$C_{I}=\left(\frac{C_{S}}{D_{N,P}^{\frac{\text{SA}}{\text{LA}}}}\right)\left(1-f+fD_{N,P}^{\frac{\text{SA}}{\text{LA}}}\right)$$ (6)

where $C_{I}$, $C_{S}$, f, and $D_{N,P}^{\text{SA}/\text{LA}}$ represent the concentration of N and P in the initial LA, the concentration of N and P in the crystallized SA (iron meteorite N and P abundance from table S3), the fraction of crystallized SA (table S3), and the partition coefficient of N and P between SA and LA calculated using Eqs. 1 and 5 of this study, respectively. f is adopted from the ranges documented in table S3.

To estimate the N and P of the bulk IMPBs, we explored two end-member differentiation regimes that could have been relevant for evolution of the IMPBs. The IMO style of differentiation is a scenario where the IMPBs melted internally (IMO) to allow metal-silicate differentiation and core formation, but there is no chemical exchange between the molten interior and the overlying atmosphere (*21*, *23*, *47*). In other words, atmophile elements do not have any opportunity to get outgassed.

The estimates for IMO were based on the following equation

$$M_{N,P}^{\text{TOT}}=M_{N,P}^{\text{CORE}}+M_{N,P}^{\text{MO}}$$ (7)

where $M_{N,P}^{\text{CORE}}$ (mass of N or P in the core) is estimated for different IMPBs in the previous section, $M_{N,P}^{\text{TOT}}$ is the total mass of N or P in the IMPB, and $M_{N,P}^{\text{MO}}$ is the mass of N or P in the silicate MO.

Similarly, for EMO or open system differentiation, there is complete melting of the planetesimal with the presence of an atmosphere overlying the molten silicate (*21*, *23*, *47*). The estimates for EMO were based on the following equation

$$M_{N}^{\text{TOT}}=M_{N}^{\text{CORE}}+M_{N}^{\text{MO}}+M_{N}^{\text{atmosphere}}$$ (8)

where $M_{N}^{\text{atmosphere}}$ is the mass of N in the atmosphere after three-reservoir fractionation. The mass of N in the atmosphere is estimated by calculating the percentage of N that gets fractionated to the atmosphere during a three-reservoir fractionation of the IMPBs. We therefore scale the IMPB N inventory accordingly to reflect this loss. The percentage of N that gets fractionated to the atmosphere for small bodies like that of the IMPBs is dependent on the size of the IMPBs. There has been considerable uncertainty regarding the sizes of the IMPBs. In addition, there are no estimates for the sizes of few of the groups (*86*, *87*). Cooling rates of various iron meteorites have suggested that the sizes of the IMPBs ranged between 5 and 300 km in diameter (*86*, *88**–**90*). Such sizes of bodies essentially fractionate around ~99 to 99.5% of the initial N inventory of IMPBs to the atmosphere within the *f*O_2_ range of IMPBs [ΔIW = −3 to −1; (*1*, *75*)].

### Estimating the P and N budgets of the BSE by accretion models

The P and N abundance in Earth’s growing mantle or MO is estimated using the mass-balance equation, where the mass of P and N is distributed between the core of the impactor and the silicate MO of Earth. This methodology has been used in previous studies (*2*, *11*, *18*). The relevant equations are as follows

$$M_{N,P}^{\text{TOT}}=M_{N,P}^{\text{CORE}}+M_{N,P}^{\text{MO}}$$ (9)

$$\text{Conc}._{N,P}^{\text{CORE},\text{MO}}=\frac{M_{N,P}^{\text{CORE,MO}}}{M^{\mathrm{CORE},\mathrm{MO}}}$$ (10)

$$\text{Conc}._{N,P}^{\mathrm{CORE}}=\text{Conc}._{N,P}^{\text{MO}}\cdot D_{N,P}^{\frac{\text{am}}{\text{sm}}}$$ (11)

where $M_{N,P}^{\text{TOT},\text{CORE},\text{MO}}$ is the mass of N or P in the entire body, core, or MO; $\text{Conc}._{N,P}^{\text{CORE},\text{MO}}$ is the concentration of N or P in the core or MO; $M^{\mathrm{CORE},\mathrm{MO}}$ is the mass of the core or MO; and $D_{N,P}^{\frac{\text{am}}{\text{sm}}}$ is the partition coefficient of N or P between alloy melt and silicate melt. The core-mass fraction of the growing Earth is fixed as 0.3. In our method, three models for the variation of *f*O_2_ during accretion were used, and the entire accretion was divided into 100 stages (*2*, *11*, *18*). The reduced model starts with the accretion of reduced material, with later accreting materials becoming more oxidized with time. In the oxidized model, the accretion begins with oxidized material, with the accreting materials becoming progressively more reduced with time. Last, the homogeneous model considers the accretion of material having a constant redox nature throughout the growth of Earth.

The equilibrium pressure used in the parameterization of N and P was equivalent to the pressure of half of the depth of the MO (final pressure, 55 GPa), and the equilibrium temperature was assumed to be a liquidus of the MO. Below 25 GPa, a pyrolitic liquidus was assumed, and above 25 GPa, a chondritic liquidus was assumed (*91**–**93*). The Ni and S contents in the alloy melt were fixed to be 5 and 1.2 wt %, respectively. The Si content in the alloy was varied from 10 to 0 wt % from low *f*O_2_ to high *f*O_2_ in the reduced model. In addition, we consider complete equilibrium of the alloy melt of the impactor’s core with that of the target’s MO. The ranges of initial P/N used for NC IMPBs, CC IMPBs, and enstatite chondrites are documented in table S6. The IMO estimates were adopted for the IMPBs because they more accurately reflect the preserved reservoirs of the differentiated building blocks. In comparison, the N-rich atmosphere produced in an EMO-like scenario would be completely lost in bodies of IMPB size, rendering their evolution more consistent with an IMO scenario.

### Choice of parameterization for estimating $D_{N,P}^{\frac{\text{am}}{\text{sm}}}$

The parameterizations of N and P partition coefficients between alloy melt and silicate melt are applied in our geochemical modeling for two purposes: (i) estimating the N and P contents of IMPBs and (ii) in the accretion model for estimating the P/N of the BSE. For N, we used the most recent parameterization from (*44*) over those in the earlier studies (*1*, *10*, *18*, *20*, *26*, *94*, *95*) because it incorporates all previous experimental data and extends to higher pressures and temperatures. To maintain consistency, we use this same parameterization for both the IMPB N budget reconstruction and terrestrial accretion model. Although a newer parameterization is available (*95*), we did not adopt it because it does not account for the effects of S and C on $D_{N,P}^{\frac{\text{am}}{\text{sm}}}$, making it unsuitable for estimating N in IMPBs. The relevant equation is

$$\begin{array}{c} \text{log}D_{N}^{\frac{\text{am}}{\text{sm}}}=a+\frac{b}{T\left(K\right)}+c\frac{P\left(\text{GPa}\right)}{T\left(K\right)}+d\text{log}\left(1-X_{C}\right)+e\text{log}\left(1-X_{S}\right)\\ +f\text{log}\left(1-X_{\text{Si}}\right)+g\text{log}\left(1-X_{O}\right)+h\left(\frac{\text{nbo}}{T}\right)+i\Delta \text{IW} \end{array}$$ (12)

where *a* = 0, *b* = 2732(156), *c* = 0, *d* = 4.04(0.60), *e* = 2.00(0.46), *f* = 5.57(0.87), *g* = 6.28(1.02), *h* = 0.49(0.03), and *i* = 0.40(0.02); *P* and *T* are the pressure and temperature of equilibration, respectively; $X_{C,S,\text{Si},O}$ are the mole fractions of C, S, Si, and O in the alloy melt; $\frac{\text{nbo}}{T}$ denotes the ratio of nonbridging oxygens (nbo) per tetrahedrally coordinated cations (T); and ΔIW is the *f*O_2_ relative to the iron-wüstite (IW) buffer.

In case of P, we use the most recent parameterization of P in (*29*). This is the latest parameterization of P partitioning between alloy melt and silicate melt and extends to high pressure and temperature ranges more applicable for the accretion of Earth. The relevant equation is

$$\text{log}D_{P}^{\frac{\text{am}}{\text{sm}}}=a+\frac{b}{T\left(K\right)}+c\frac{P\left(\text{GPa}\right)}{T\left(K\right)}+d\left(\frac{\text{nbo}}{T}\right)-\frac{5}{4}\times \Delta \text{IW}$$ (13)

where *a* = −3.12(171), *b* = 3835(2860), *c* = 594(112), and *d* = −0.650(103) for *P* < 15 GPa and *a* = 4.27(72), *b* = −1464(883), *c* = −212(49), and *d* = −1.19(15) for *P* > 15 GPa.

## Supplementary Materials

### This PDF file includes:

Supplementary Text Figs. S1 to S10 Tables S1 to S6 References
