# Supplementary material for: Phosphorus-nitrogen systematics of first-generation planetesimals constrain life-essential element delivery to Earth
Source: Sci Adv. 2026 Jun 3;12(23):eaed8749. doi: 10.1126/sciadv.aed8749 (PMC13232560; doi:10.1126/sciadv.aed8749)
Supplement: Supplementary file 1 — Supplementary Text Figs. S1 to S10 Tables S1 to S6 References [file sciadv.aed8749_sm.pdf]

Science Advances
AAAS

Supplementary Materials for

Phosphorus-nitrogen systematics of first-generation planetesimals constrain life-essential element delivery to Earth

Debjeet Pathak *et al.*

Corresponding author: Debjeet Pathak, dp55@rice.edu

Sci. Adv. **12**, eaed8749 (2026)
DOI: 10.1126/sciadv.aed8749

This PDF file includes:

Supplementary Text
Figs. S1 to S10
Tables S1 to S6
References

Supplementary Text

Phase Relations in the Fe-P and Fe-S-P Systems: Comparison with previous studies

The phase relations observed in our experiments are consistent with previous studies. In the S-free, Fe-P system, we observe a slight decrease in the P content in the liquid alloy from ~10.1 wt.% to ~7.7 wt.% with increasing temperature, similar to the experimental study of ref. *(41)*. Similarly, for the solid alloy we observe an increase in the P content of the solid alloy with increasing temperature. We don't observe the phase Fe_2P as seen in some experiments of ref. *(41)*. This can be attributed to the fact the Fe_2P-bearing experiments in ref. *(41)* were conducted at a bulk P content of greater than 18.8 wt.%. However, none of our experiments were conducted at such high P content.

In the Fe-S-P system, we observed only solid alloy and liquid alloy in our experiments for temperature >1075 °C. In experiments having an equilibrium temperature less than 1075 °C and having a bulk composition rich in S and P (Fe-S1-P12, Fe-S6-P10) we obtain schreibersite and troilite. This is consistent with previous experiments (*40*) where troilite and schreibersite are observed for experiments at a temperature and pressure of 1000 °C and 3 GPa and having a bulk starting composition of Fe-2S-8P. Similarly, in the same study (*40*) and for the same bulk composition and pressure, experiment at 1100 °C shows absence of troilite and schreibersite. However, in this study, experiments at 5 GPa-1000 °C, and of the same starting mix (Fe-2S-8P) don't generate troilite and schreibersite (ref. *40*). Only four of our experiments are super liquidus. We observe a completely molten liquid alloy at a temperature of 1250 °C for a starting mix of Fe-S1-P12. In addition, we observe only liquid alloy for experiments at 1200°C and 1600°C for the starting mix Fe-S5-P6-N0.5-Ni8. Similarly, the composition Fe–S15–P1–N0.5–Ni8 produced only liquid alloy at 1600°C. Furthermore, our super liquidus experiments do not show any signs of liquid immiscibility.

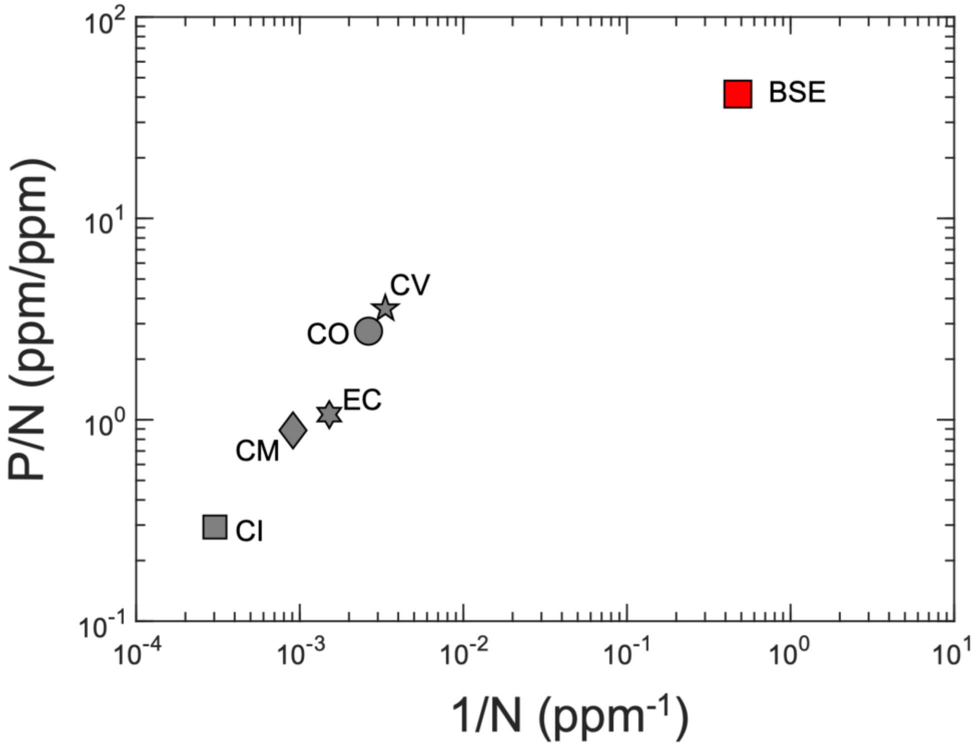

Figure S1. **The N and P contents of different chondrites and that of the bulk silicate Earth (BSE) in the1/N (ppm^{-1}) vs P/N (ppm/ppm) space.** We observe a diagonal trend signifying almost negligible variation of P with significant variation of N content among the various chondrite groups. This negligible variation of P compared to N shows the relative inertness of P to parent body processes like thermal metamorphism and aqueous alteration. Moreover, none of the estimates of chondrites matches the BSE signature. N estimates are from refs. (*49, 97*) and P estimates are from refs. (*66, 96*).

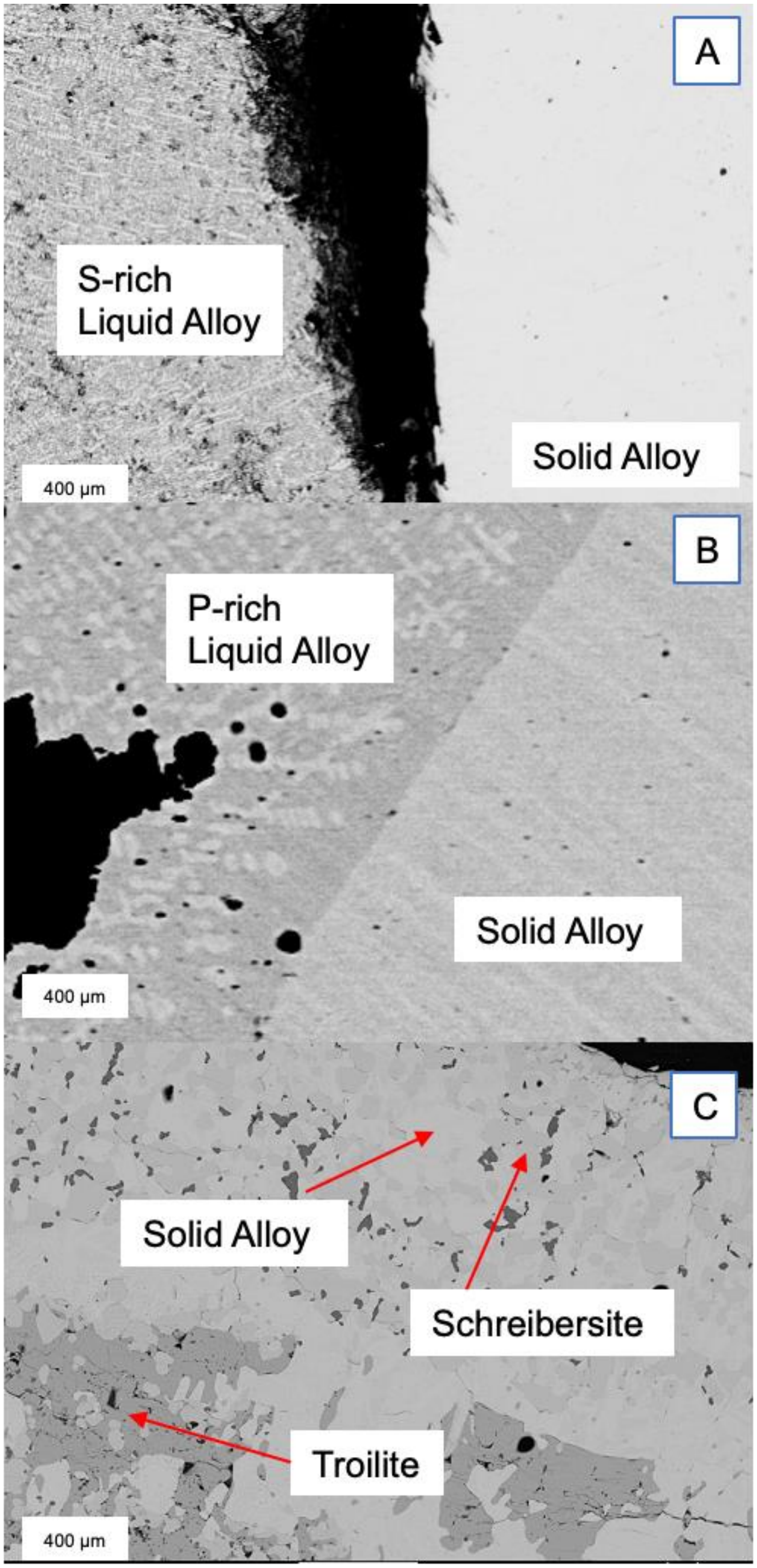
A
S-rich
Liquid Alloy
Solid Alloy
400 µm
B
P-rich
Liquid Alloy
Solid Alloy
400 µm
C
Solid Alloy
Schreibersite
Troilite
400 µm

Figure S2. **Representative back-scattered electron images of experimental products from this study. (A)** Experiment G824 showing S-free, P-bearing quenched liquid alloy (LA) coexisting with Fe-rich solid alloy (SA). **(B)** An experiment (G785) with P-bearing solid alloy (SA) and liquid alloy (LA). **(C)** Schreibersite (Scb.), troilite (Tro.), and solid alloy (SA) in the experiment G820.

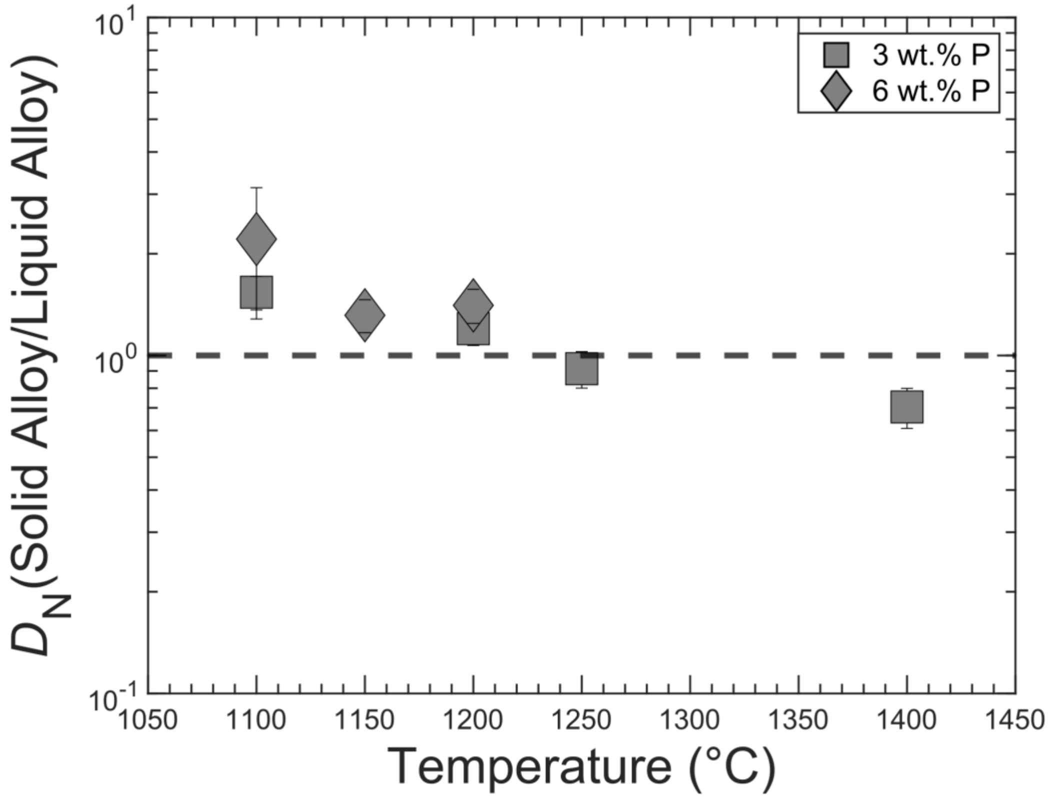

Figure S3. $\boldsymbol{D_{\mathrm{N}}^{\mathrm{SA/LA}}}$ **for two sets of S-free experiments having 3 and 6 wt.% initial bulk P plotted against temperature.** $D_{\mathrm{N}}^{\mathrm{SA/LA}}$ decreases slightly with increasing temperature with N becoming mildly incompatible at ≥~1250 °C.

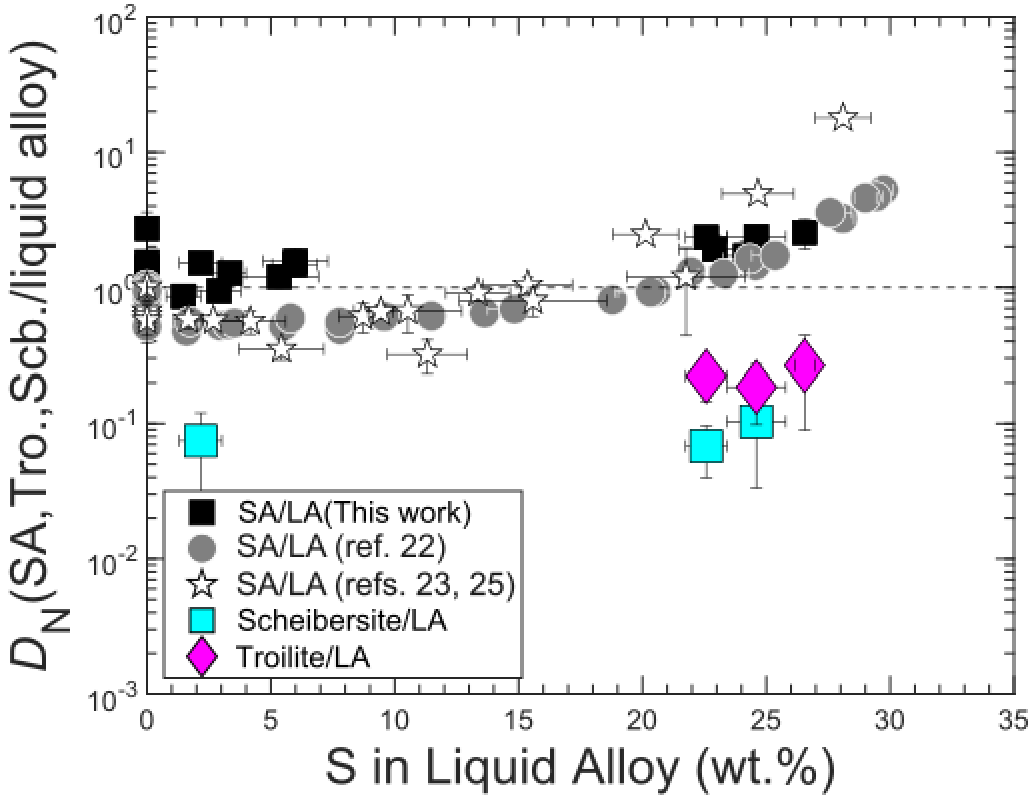

Figure S4. $\boldsymbol{D_{\mathrm{N}}^{\mathrm{SA/LA}}}$ **,** $\boldsymbol{D_{\mathrm{N}}^{\mathrm{Tro./LA}}}$ **(Tro. – Troilite),** $\boldsymbol{D_{\mathrm{N}}^{\mathrm{Scb./LA}}}$ **(Scb. – Schreibersite) plotted against the S content of the liquid alloy.** $D_{\mathrm{N}}^{\mathrm{Scb./LA}}$, $D_{\mathrm{N}}^{\mathrm{Tro./LA}}$ are an order of magnitude lower than $D_{\mathrm{N}}^{\mathrm{SA/LA}}$ for the entire range of S concentration in the liquid alloy. Interestingly, $D_{\mathrm{N}}^{\mathrm{Tro./LA}}$ is slightly higher than $D_{\mathrm{N}}^{\mathrm{Scb./LA}}$. Only a mild dependence of $D_{\mathrm{N}}^{\mathrm{Scb./LA}}$ on the S content of the liquid alloy is observed.

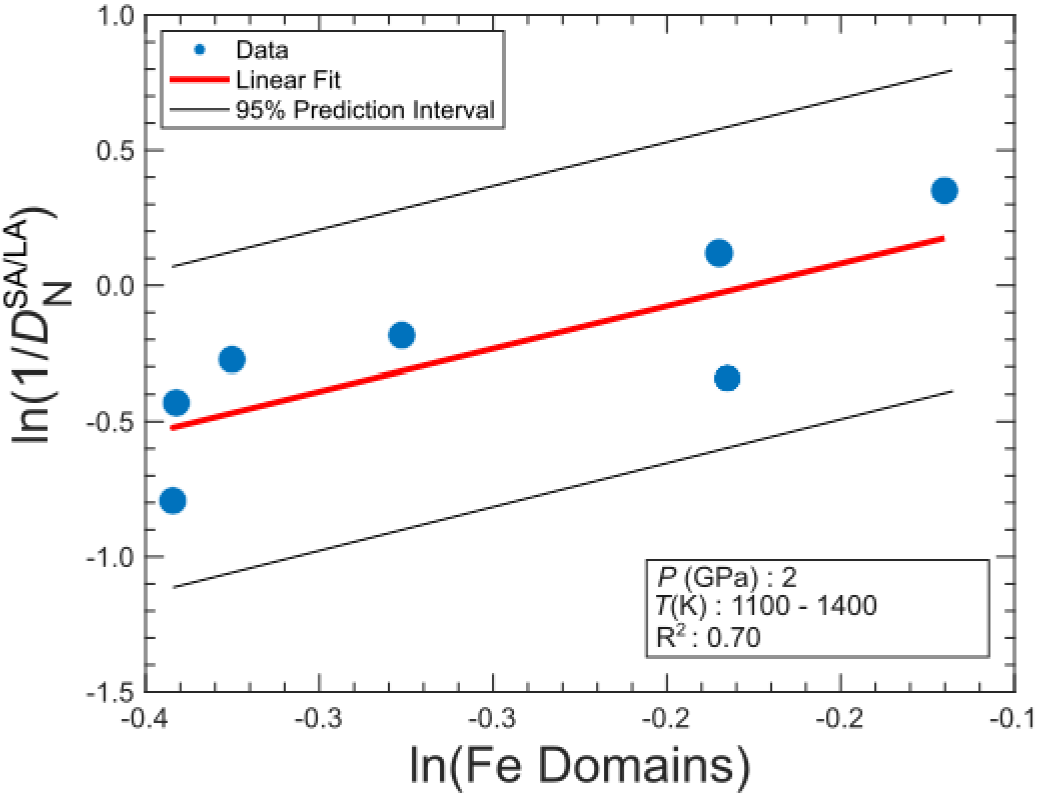

Figure S5. **Parameterization of N partition coefficient between Fe-rich solid alloy and S-free, P-bearing liquid alloy, $D_N^{SA/LA}$, following the approach of ref. (*85*).**

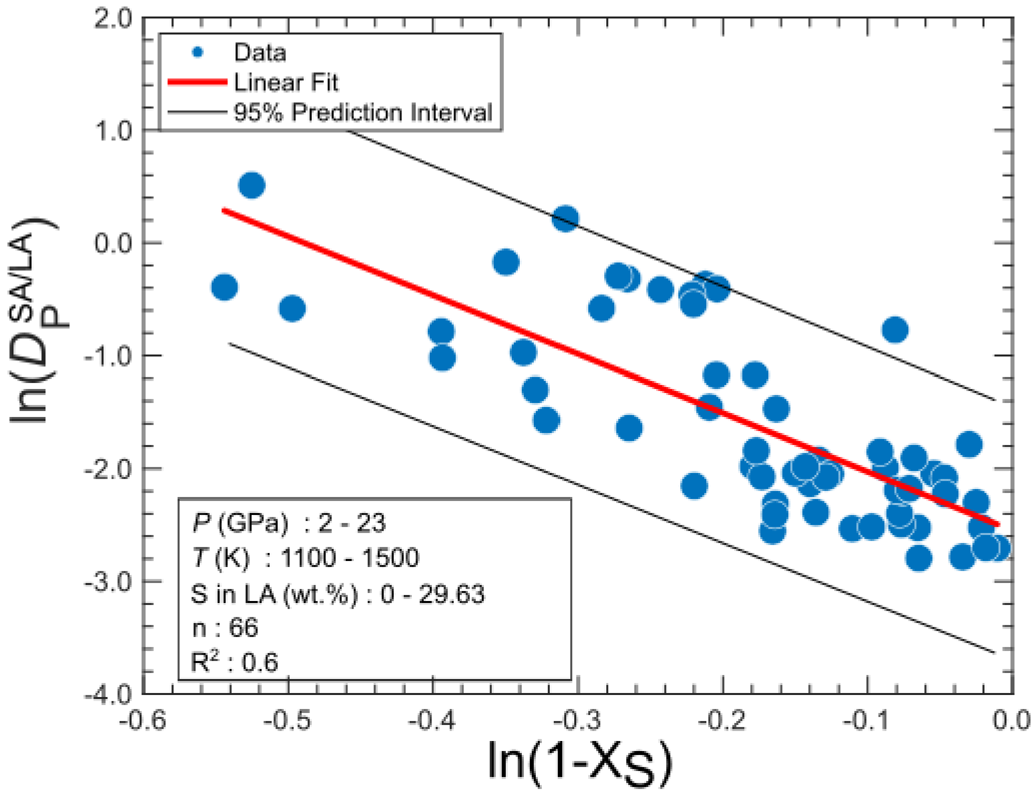

Figure S6. **Parameterization of phosphorus partition coefficient between Fe-rich solid alloy and S, P-bearing liquid alloy, $\boldsymbol{D_{\mathrm{P}}^{\mathrm{SA/LA}}}$, following the approach of ref. (*40*).**

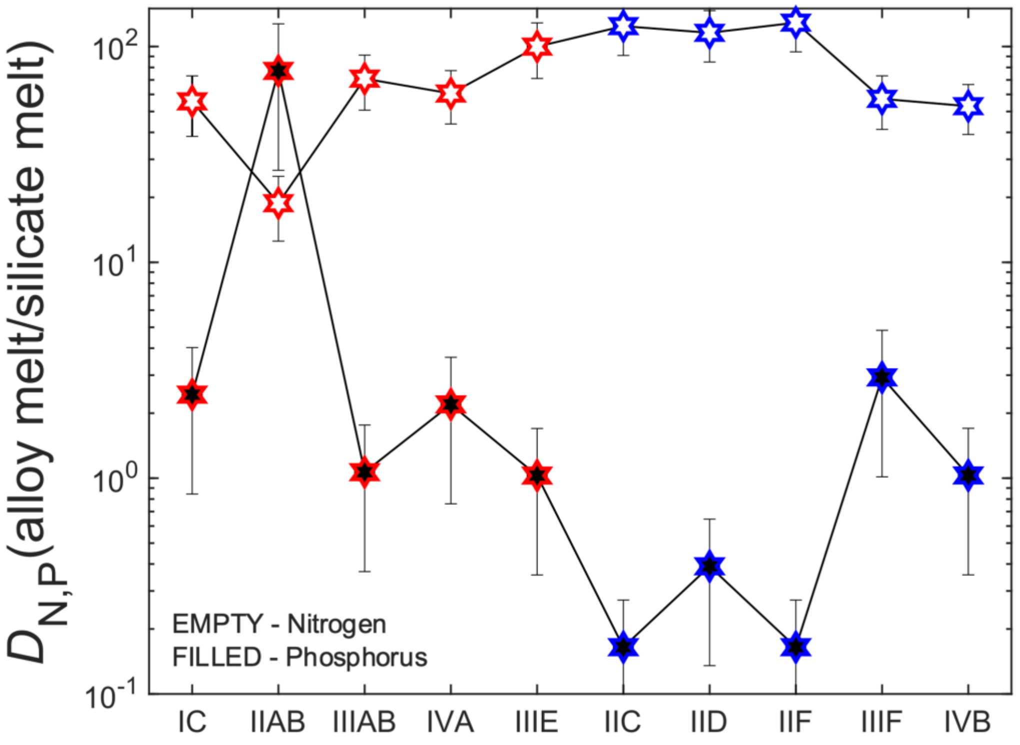

Figure S7. **Alloy melt/silicate melt partition coefficients for P and N estimated for different iron meteorite groups and documented in Table S5.** The P and N partition coefficients are estimated using the parameterization in ref. (*29*) and ref. (*44*), respectively.

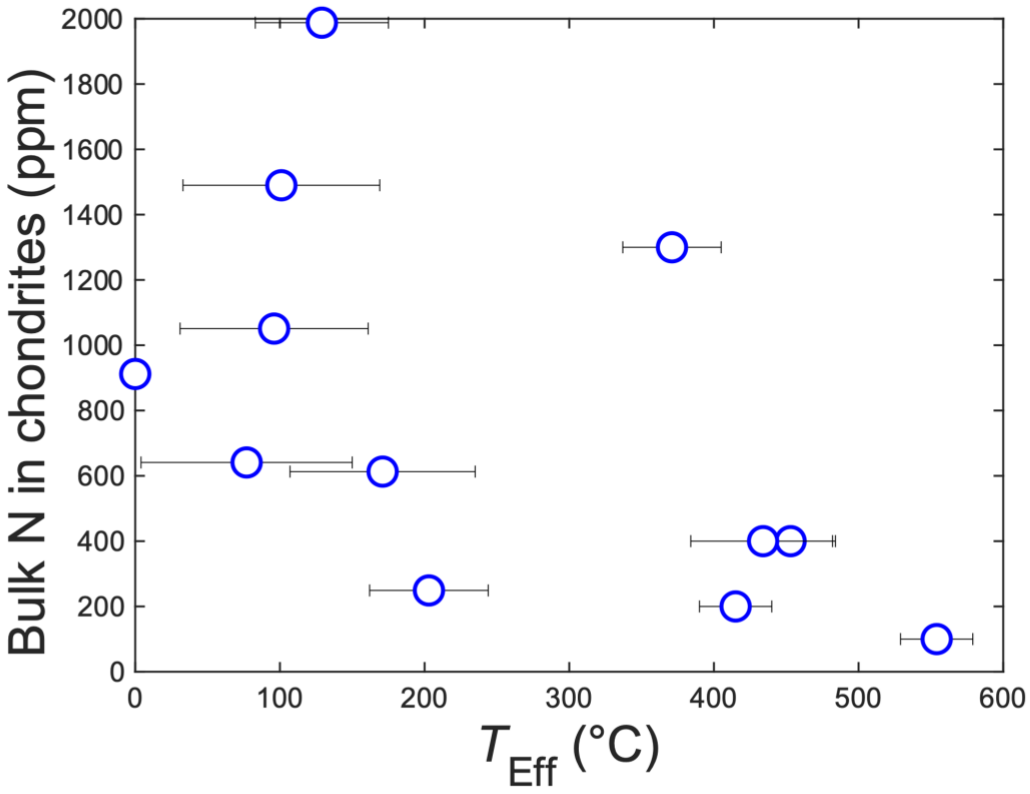

Figure S8. **Bulk N in chondrite (ref. *49*) plotted against the effective metamorphic temperature (T_{Eff}) estimated from the organic thermometer (ref. *50*).** N content decreases mostly by a factor of ~3-5 but not exceeding a factor of 10 with increasing temperature.

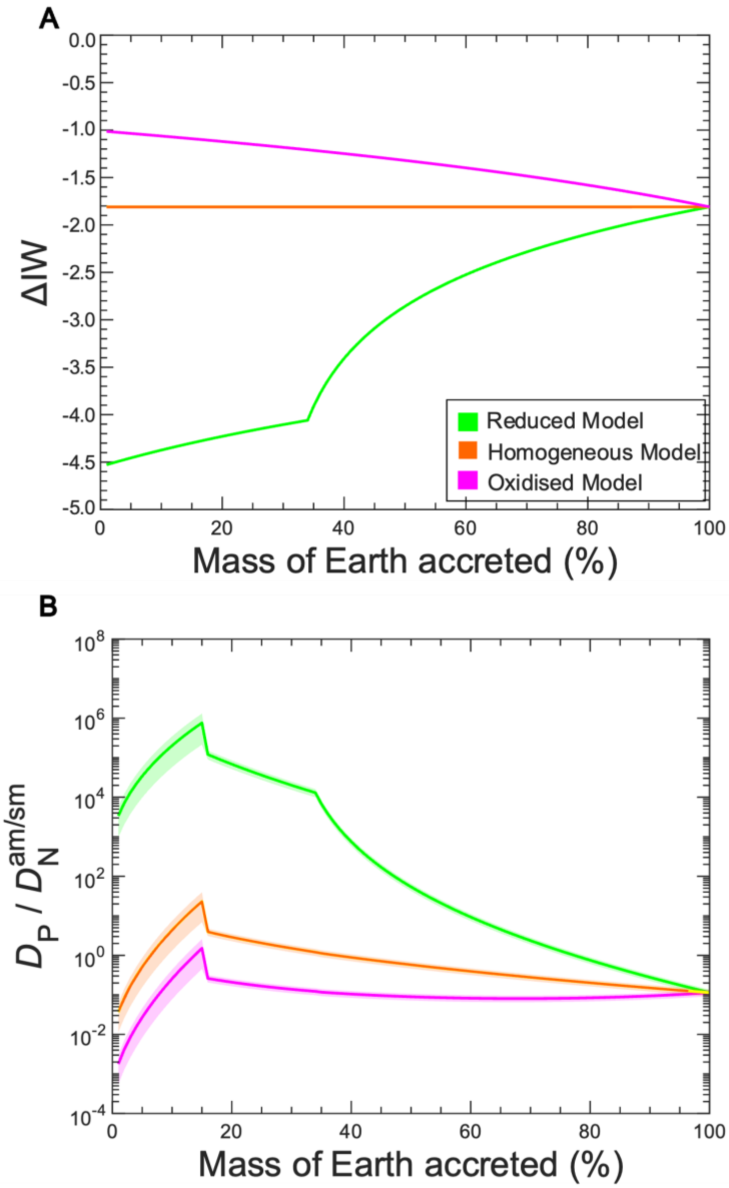

Figure S9. **Redox evolution and $D_{\mathbf{P}}^{\mathbf{am/sm}}/D_{\mathbf{N}}^{\mathbf{am/sm}}$ plotted against the percentage of Earth's mass accreted. (A)** The redox evolution plotted against the mass fraction of Earth accreted (in % terms) used in our model, i.e., the *reduced* model, *oxidized* model, and the *homogenous* model. **(B)** $D_{\mathrm{P}}^{\mathrm{am/sm}}/D_{\mathrm{N}}^{\mathrm{am/sm}}$ for the three accretion models are plotted against the mass fraction of Earth accreted (in % terms). The shaded region in **(B)** represents the uncertainty which has been

estimated by propagating the uncertainties in the partition coefficient of both P and N using the model of ref. for N (*44*) and P (*29*).

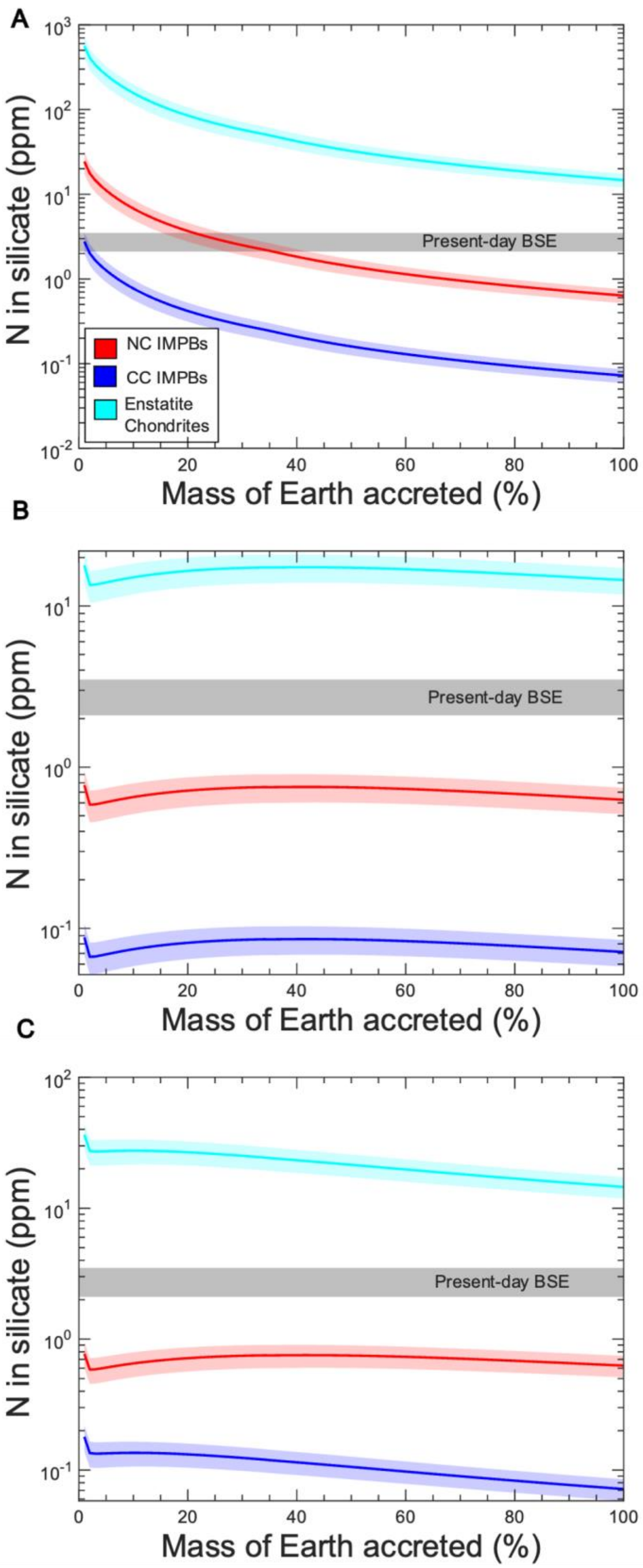

A
B
C
N in silicate (ppm)
Mass of Earth accreted (%)
Present-day BSE
NC IMPBs
CC IMPBs
Enstatite
Chondrites

Figure S10. **N (ppm) budget in the silicate magma ocean as a function of the percentage of Earth's mass accreted for three different N budgets in the building blocks corresponding to that of the NC IMPBs, CC IMPBs, and enstatite chondrites.** The grey band represents the present-day BSE estimate (refs. *66, 97*). The shaded regions along the curves represent uncertainty in the modeled values, estimated by propagating uncertainties in the estimated partition coefficient $D_{\mathrm{N}}^{\mathrm{am/sm}}$. Calculations were performed for three redox evolution pathways: (A) reduced—accretion progressing from reduced to more oxidized material; (B) oxidized—accretion progressing from oxidized to more reduced material; and (C) homogeneous—accretion of material with similar redox characteristics throughout growth (given in Fig. S9). Across all redox pathways, accretion dominated by NC IMPBs provides the closest match to the present-day N budget of the BSE, yielding N abundances depleted by approximately a factor of ~3.5 relative to current estimates. In contrast, CC IMPBs produce BSE N budgets depleted by at least one order of magnitude, whereas accretion of enstatite chondrites results in N abundances enriched by less than one order of magnitude relative to the present-day BSE inventory.

Table S1. Summary of experiments and obtained partition coefficients of nitrogen and phosphorus between Fe-rich solid alloy and liquid alloy, schreibersite and liquid alloy, and troilite and liquid alloy.

Expt. No.[a]	*T* (°C)	*P* (GPa)	Time (h)	Starting Composition[Δ]	Assemblages	$D_N^{SA/LA}$	$D_N^{Scb./LA}$	$D_N^{Tro./LA}$	$D_P^{SA/LA}$
B657	1100	2	28	Fe88.5-P3-S0-N0.5-Ni8	SA+LA	1.54(17)			0.12(00)
G785	1200	2	24	Fe88.5-P3-S0-N0.5-Ni8	SA+LA	1.20(23)			0.13(02)
T353_2+	1250	2	18	Fe88.5-P3-S0-N0.5-Ni8	SA+LA	0.91(11)			0.13(01)
T348_2+	1400	2	2	Fe88.5-P3-S0-N0.5-Ni8	SA+LA	0.70(10)			0.11(01)
B701	1100	2	28	Fe85.5-P6-S0-N0.5-Ni8	SA+LA	2.73(0.87)			0.16(05)
B700	1150	2	20	Fe85.5-P6-S0-N0.5-Ni8	SA+LA	1.32(15)			0.13(01)
B659	1200	2	9	Fe85.5-P6-S0-N0.5-Ni8	SA+LA	1.41(16)			0.13(01)
T319	1200	2	6	Fe84.7-P3-S0.8-N0.5-Ni8	SA+LA	0.86(11)			0.10(01)
B658	1100	2	26	Fe87.5-P3-S1-N0.5-Ni8	SA+LA	1.27(21)			0.13(00)
T347	1200	2	12	Fe87.5-P3-S1-N0.5-Ni8	SA+LA	0.94(11)			0.12(01)
B643	1250	2	26	Fe87.5-P3-S1-N0.5-Ni8	SA+LA	1.20(18)			0.14(02)
G820	1025	2	24	Fe86.5-P12-S1-N0.5	SA+LA+ Scb.+Tro.	2.34(35)	0.07(03)	0.22(08)	0.46(08)
T350	1075	2	16	Fe86.5-P12-S1-N0.5	SA+LA+ Scb.	1.52(13)	0.07(05)		0.08(01)
T353_1+	1250	2	18	Fe86.5-P12-S1-N0.5	LA				
T346	1050	2	36	Fe81.5-P6-S5-N0.5-Ni8	SA+LA	1.47(18)			0.16(04)
T340	1100	2	15	Fe81.5-P6-S5-N0.5-Ni8	SA+LA	1.56(38)			0.15(03)
T341	1200	2	40	Fe81.5-P6-S5-N0.5-Ni8	LA				
T342	1600	2	2	Fe81.5-P6-S5-N0.5-Ni8	LA				
G825	1050	2	26	Fe81.5-P1-S10-N0.5-Ni8	SA+LA+Tro.	2.55(0.63)		0.27(18)	1.24(1.0)
G824	1100	2	30	Fe81.5-P1-S10-N0.5-Ni8	SA+LA	1.95(58)			0.70(15)
G826	1125	2	28	Fe81.5-P1-S10-N0.5-Ni8	SA+LA	1.71(47)			0.74(18)
T352	1025	2	40	Fe75.5-P6-S10-N0.5-Ni8	SA+LA+Scb .+Tro.	2.37(32)	0.10(07)	0.19(09)	0.56(14)
B703	1600	2	1	Fe75.5-P1-S15-N0.5-Ni8	LA				

Abbreviations: - SA - Solid Alloy, LA - Liquid Alloy, Scb. - Schreibersite, Tro. - Troilite.

Δ-Starting compositions are noted in weight percent of elements; Fe88.5-P3-S0-N0.5-Ni8 should be read as 88.5 wt.% Fe, 3 wt.% P, 0 wt.% S, 0.5 wt.% N, 8 wt.% Ni.

1σ error for $D_{\mathrm{N}}^{SA/LA}$, $D_{\mathrm{N}}^{(Scb./Tro.)/LA}$ and $D_{\mathrm{P}}^{SA/LA}$ is obtained by propagating 1σ deviation error on N content in the solid alloy, schreibersite, troilite and liquid alloy. Errors in parentheses are 1σ of the mean, reported as least units cited; for example, 1.54 (17) should be read as 1.54 ± 0·17.

[a] The experiments are arranged in increasing order of S content in the starting mix. Thereafter in each group of fixed S content, the experiments are arranged in increasing order of temperature.

[+] Denotes experiment run in double chambered MgO capsules.

Table S2. The composition of experimental phases.

Expt. No.	Phase (n)	Fe (wt.%)	Ni (wt.%)	P (wt.%)	S (wt.%)	N (wt.%)	O (wt.%)	Total (wt.%)
B657	SA (16)	89.71	8.01	1.11	0.00	0.46	0.14	99.44
	1σ	0.35	0.10	0.02	0.00	0.03	0.06	0.34
	LA (15)	80.62	9.34	9.25	0.01	0.30	0.16	99.69
	1σ	0.39	0.11	0.16	0.00	0.03	0.04	0.46
G785	SA (21)	89.57	7.79	1.04	0.00	0.40	0.19	98.99
	1σ	0.49	0.10	0.02	0.00	0.06	0.02	0.57
	LA (21)	81.36	8.96	8.27	0.03	0.34	0.17	99.13
	1σ	0.64	0.16	0.69	0.01	0.04	0.03	0.45
T353_2+	SA (13)	91.70	7.98	0.86	0.00	0.40	0.12	101.06
	1σ	0.19	0.05	0.02	0.00	0.02	0.05	0.21
	LA (15)	84.59	8.96	6.72	0.01	0.44	0.15	100.86
	1σ	0.49	0.12	0.46	0.00	0.05	0.05	0.25
T348	SA (15)	90.02	7.65	0.54	0.01	0.36	0.30	98.88
	1σ	0.25	0.06	0.02	0.00	0.04	0.04	0.28
	LA (16)	84.67	8.32	4.91	0.00	0.52	0.34	98.76
	1σ	0.84	0.13	0.77	0.05	0.04	0.12	0.32
B701	SA (15)	91.33	7.05	1.38	0.00	0.59	0.33	100.68
	1σ	0.60	0.11	0.39	0.00	0.04	0.04	0.50
	LA (7)	81.65	9.29	9.44	0.00	0.27	0.42	101.06
	1σ	2.54	0.87	2.55	0.00	0.11	0.16	0.37
B700	SA (15)	91.12	7.39	1.15	0.00	0.54	0.27	100.47
	1σ	0.22	0.07	0.07	0.00	0.03	0.03	0.21
	LA (16)	82.09	8.63	9.20	0.01	0.41	0.33	100.67
	1σ	0.39	0.29	0.43	0.00	0.04	0.08	0.20
B659	SA (17)	90.54	7.42	1.15	0.00	0.60	0.26	99.97
	1σ	0.53	0.12	0.03	0.00	0.04	0.03	0.57
	LA (18)	82.74	8.54	8.97	0.01	0.43	0.25	100.93
	1σ	0.64	0.20	0.62	0.00	0.04	0.03	0.31
T319	SA (12)	89.45	7.48	0.57	0.02	0.51	0.30	98.33
	1σ	0.66	0.07	0.02	0.00	0.03	0.11	0.62
	LA (10)	81.08	8.84	5.70	1.47	0.59	0.50	98.19
	1σ	1.54	0.35	0.85	0.70	0.07	0.15	1.32
B658	SA (15)	90.07	7.77	0.97	0.02	0.47	0.25	99.54
	1σ	0.33	0.08	0.03	0.00	0.03	0.09	0.32
	LA (18)	79.14	9.04	7.46	3.34	0.37	0.49	99.84

	1σ	0.59	0.25	0.44	0.66	0.05	0.17	0.29
T347	SA (15)	91.35	7.71	0.81	0.02	0.42	0.26	100.56
	1σ	0.27	0.07	0.02	0.00	0.02	0.03	0.31
	LA (13)	82.56	8.68	6.47	2.90	0.44	0.29	101.33
	1σ	0.92	0.19	0.63	0.88	0.04	0.09	0.35
B643	SA (16)	90.49	7.20	0.63	0.03	0.50	0.33	99.19
	1σ	0.49	0.06	0.02	0.00	0.06	0.04	0.52
	LA (16)	79.47	8.02	4.64	5.34	0.41	0.68	98.57
	1σ	1.34	0.22	0.54	1.58	0.04	0.10	0.41
G820	SA (16)	90.20	8.10	0.67	0.01	1.05	0.27	100.30
	1σ	0.20	0.10	0.02	0.00	0.06	0.04	0.25
	LA (16)	67.76	7.98	1.47	22.58	0.45	0.46	100.69
	1σ	0.78	0.48	0.26	0.84	0.06	0.15	0.21
	Scb. (15)	76.64	8.97	15.37	0.57	0.03	0.26	101.77
	1σ	0.15	0.07	0.16	0.02	0.01	0.06	0.31
	Tro. (13)	62.40	0.62	0.00	34.71	0.10	1.23	98.89
	1σ	0.88	0.03	0.00	0.71	0.03	0.56	1.21
T350	SA (14)	97.09	0.01	0.69	0.02	1.36	0.24	99.41
	1σ	0.32	0.01	0.03	0.04	0.04	0.04	0.33
	LA (15)	88.45	0.02	8.66	2.17	0.89	0.21	100.41
	1σ	0.59	0.02	0.62	0.86	0.07	0.03	0.26
	Scb. (14)	84.50	0.01	15.35	0.49	0.07	0.29	100.71
	1σ	0.52	0.01	0.61	0.12	0.04	0.06	0.24
T353_1+	LA	87.75	0.00	11.94	0.94	0.48	0.12	101.23
	1σ	0.26	0.00	0.19	0.08	0.05	0.03	0.12
T346	SA (15)	90.59	7.27	1.11	0.12	0.53	0.34	99.94
	1σ	0.66	0.09	0.26	0.20	0.04	0.02	0.26
	LA (11)	78.15	8.69	7.05	5.97	0.36	0.42	100.64
	1σ	0.78	0.21	0.31	0.91	0.03	0.13	0.26
T340	SA (16)	90.74	6.94	0.92	0.05	0.58	0.25	99.48
	1σ	0.47	0.05	0.12	0.08	0.03	0.06	0.28
	LA (12)	78.44	8.24	6.18	5.99	0.46	0.37	99.68
	1σ	0.98	0.37	0.83	1.31	0.05	0.09	0.71
T341	LA (15)	80.50	8.15	5.76	5.09	0.50	0.34	100.34
	1σ	1.24	0.25	0.68	1.52	0.06	0.07	0.32
T342	LA (15)	81.29	8.25	5.92	4.93	0.51	0.51	101.41
	1σ	1.79	0.45	0.76	1.95	0.05	0.11	0.31
G825	SA (16)	89.17	8.85	0.58	0.02	0.54	0.29	99.45
	1σ	0.27	0.14	0.02	0.00	0.05	0.03	0.31
	LA (16)	65.03	7.44	0.47	26.55	0.21	0.56	100.26

	1σ	0.63	0.64	0.11	0.40	0.05	0.12	0.24
	Tro. (15)	62.82	0.61	0.00	35.70	0.05	0.51	99.69
	1σ	0.25	0.04	0.00	0.16	0.03	0.14	0.38
G824	SA (16)	89.61	8.14	0.44	0.02	0.57	0.23	99.00
	1σ	0.36	0.08	0.02	0.00	0.04	0.04	0.36
	LA (12)	66.14	7.81	0.62	23.14	0.29	1.01	99.01
	1σ	2.09	0.76	0.13	1.60	0.08	0.26	0.33
G826	SA (15)	90.60	8.50	0.46	0.02	0.56	0.17	100.31
	1σ	0.23	0.08	0.02	0.00	0.04	0.05	0.23
	LA (16)	66.77	7.60	0.63	23.96	0.31	1.01	100.27
	1σ	1.82	0.87	0.16	1.64	0.09	0.66	0.94
T352	SA (16)	88.98	8.35	0.67	0.01	1.10	0.50	99.61
	1σ	0.39	0.07	0.03	0.00	0.05	0.12	0.34
	LA (16)	65.91	7.73	1.20	24.60	0.46	0.62	100.52
	1σ	0.37	0.88	0.30	1.19	0.06	0.08	0.25
	Scb. (15)	74.78	8.89	15.08	0.55	0.04	0.98	100.32
	1σ	0.60	0.11	0.21	0.02	0.03	0.44	0.46
	Tro. (15)	62.47	0.64	0.00	35.56	0.09	0.74	99.49
	1σ	0.22	0.04	0.00	0.58	0.04	0.17	0.59
B703	LA (12)	71.91	7.49	1.01	17.43	0.43	1.49	99.75
	1σ	3.17	1.03	0.22	2.60	0.07	0.89	1.15

SA – Solid Alloy, LA-Liquid Alloy, Scb – Schreibersite, Tro. - Troilite

n represents the number of obtained measurements in EPMA that were averaged for the reported data.

The number of analyses reported in Table S1. were randomly analyzed to check sample phase homogeneity.

+ Experiments were performed in double chambered capsules.

Table S3. Range of input parameters used in the calculation of N and P budget of IMPB cores.

	N (ppm) [a]	P (wt.%) [+]	S (wt.%) *	C (wt.%) [#]	f [b]
**NC**					
IC	15.10-67.50	0.20-0.39	15.00	0.111	0.01-0.27
IIAB	1.63-59.00	0.20-0.90	15.00	0.004	0.03-0.56
IIIAB	1.60-52.60	-	9.00	0.009	0.30-0.48
IIIE	8.50-88.90	0.15-0.31	7.00	0.004	0.30
IVA	0.28-34.00	-	3.00	0.0003	0.40-0.75
**CC**					
IIC	4.10-16.90	0.30-0.48	6.00	0.03	0.10-0.26
IID	16.30-44.00	0.30-0.90	0.10	0.04	0.20-0.70
IIF	15.70-23.70	0.10-0.30	5.00	0.006	0-0.30
IIIF	1.40-9.00	-	2.00	0.003	0-0.72
IVB	0.40-2.10	0.05-0.19	0.10	0.0004	0-0.78

[a] Measured N concentrations (in ppm) in iron meteorites are from the compilation of ref. (*9*), which was taken from – ref. (*37*) - IIIAB; ref. (*36*) - IC, IIAB, IIIAB, IIC, IID, IIF, IIIE, IIIF, IVB; ref. (*38*) - IIAB, IIIAB, IIIF, IVA, IVB; ref. (*35*)- IC, IIAB, IIIAB, IIC, IID, IIF, IVB; ref. (*98*)– IC, IIAB, IIC, IID, IIF, IIIAB, IIIE, IIIF, IVA, IVB; ref. (*99*) – IC, IIAB, IIIAB, IVA; ref. (*39*) - IID, IIAB, IIIAB, IVA, IVB; ref. (*100*) – IIIE; ref. (*101*) - IVA, IVB;

[b] Extent of crystallization of the iron meteorite cores, f are our used inputs based upon the fractional crystallization models estimates of various siderophile elements - IIC- ref. (*102*), IID- ref. (*46, 103*), IIIF- ref. (*32*), IIF- ref. (*46*), IVB - ref. (*32*), IC- ref. (*104*), IIAB- ref. (*105*), IIIAB – ref. (*106*), IVA – ref. (*107*), IIIE – ref. (*108*)

\+ P concentrations are from ref. (*33*).

* S concentrations are from ref. (*33*)

[#] Estimated C concentrations (in ppm) in the completely molten bulk core are from ref. (*22*)

NC – Non- Carbonaceous
CC – Carbonaceous

Table S4. Estimates of nitrogen and phosphorus partition coefficients between alloy melt and silicate melt

	$D_N^{am/sm}$ [a]	1σ	$D_P^{am/sm}$ [b]	1σ	CMF[c]
**NC**					
IC	55.70	17.37	2.43	1.59	0.21
IIAB	18.78	6.24	77.05	50.36	0.22
IIIAB	70.95	20.25	1.06	0.69	0.21
IIIE	99.86	28.76	1.02	0.67	0.21
IVA	60.54	16.77	2.10	1.44	0.19
**CC**					
IIC	124.30	33.39	0.16	0.11	0.18
IID	115.73	30.96	0.39	0.26	0.14
IIF	128.99	34.49	0.16	0.11	0.14
IIIF	57.21	15.91	2.92	1.91	0.18
IVB	52.89	13.76	1.02	0.67	0.04

[a] $D_N^{am/sm}$ is estimated from ref. (*44*).
[b] $D_P^{am/sm}$ is estimated from ref. (*29*).
[c] CMF = Core-Mass Fraction is from ref. (*46*).

Table S5. Modeled estimates of nitrogen and phosphorus contents in the bulk cores of IMPBs in this study.

	N in bulk core (ppm; This study)*	1σ[a]	P in bulk core (ppm; This study)*	1σ[a]	P in bulk core (FC; ppm)[+]
<u>NC</u>					
IC	32.65	0.16	6602	32	4900
IIAB	16.15	0.12	6250	39	6000
IIIAB	28.58	0.21	-	-	3000
IVA	18.56	0.14	-	-	1200
IIIE	60.79	0.38	6333	25	5000
<u>CC</u>					
IIC	11.47	0.06	21394	63	22000
IID	37.13	0.15	34366	194	19000
IIF	27.71	0.05	12600	60	7000
IIIF	6.81	0.04	-	-	12000
IVB	1.94	0.01	8745	62	4700

[a] Standard error of the median
* Estimated by the batch crystallization model of this study.
+ Most preferred value as selected from ref. (*33*), which represents the estimates by fractional crystallization model.
NC – Non- Carbonaceous
CC – Carbonaceous
- No P estimate in this work for IIIAB, IIIF, IVA as they do not have literature estimate of P.

Table S6. Initial N and P budget for the Earth's accretional model

	N (ppm)	1σ	P (ppm)	1σ
NC IMPBs	28.58*	2.87#	1399.12*	622.86 #
CC IMPBs	3.25*	1.68#	43014.01*	14663.83 #
Enstatite Chondrites	660[a]	300	1585[b]	580

* Represents the median value of all the IMPBs
# Represents the standard error of the median
[a] – ref. (*68*)
[b] – ref. (*96*)

REFERENCES

1. D. S. Grewal, R. Dasgupta, T. Hough, A. Farnell, Rates of protoplanetary accretion and differentiation set nitrogen budget of rocky planets. *Nat. Geosci.* **14**, 369–376 (2021).

2. R. Dasgupta, D. S. Grewal, Origin and early differentiation of carbon and associated life-essential volatile elements on Earth. *Deep Carbon* , 4–39 (2019).

3. F. Spitzer, C. Burkhardt, G. Budde, T. S. Kruijer, A. Morbidelli, T. Kleine, Isotopic evolution of the inner solar system inferred from molybdenum isotopes in meteorites. *Astrophys. J. Lett.* **898**, L2 (2020).

4. N. Sugiura, W. Fujiya, Correlated accretion ages and ε54Cr of meteorite parent bodies and the evolution of the solar nebula. *Meteorit. Planet. Sci.* **49**, 772–787 (2014).

5. C. M. O. D. Alexander, R. Bowden, M. L. Fogel, K. T. Howard, C. D. K. Herd, L. R. Nittler, The provenances of asteroids, and their contributions to the volatile inventories of the terrestrial planets. *Science* **337**, 721–723 (2012).

6. M. E. Newcombe, S. G. Nielsen, L. D. Peterson, J. Wang, C. M. O. D. Alexander, A. R. Sarafian, K. Shimizu, L. R. Nittler, A. J. Irving, Degassing of early-formed planetesimals restricted water delivery to Earth. *Nature* **615**, 854–857 (2023).

7. C. M. O. D'Alexander, The origin of inner Solar System water. *Philos. Trans. A. Math. Phys. Eng. Sci/* **375**, 20150384 (2017).

8. W. Liu, Y. Zhang, F. L. H. Tissot, G. Avice, Z. Ye, Q. Z. Yin, I/Pu reveals Earth mainly accreted from volatile-poor differentiated planetesimals. *Sci. Adv.* **9**, eadg9213 (2023).

9. D. S. Grewal, R. Dasgupta, B. Marty, A very early origin of isotopically distinct nitrogen in inner Solar System protoplanets. *Nat. Astron.* **5**, 356–364 (2021).

10. D. S. Grewal, R. Dasgupta, C. Sun, K. Tsuno, G. Costin, Delivery of carbon, nitrogen, and sulfur to the silicate Earth by a giant impact. *Sci. Adv.* **5**, eaau3669 (2019).

11. Y. Li, R. Dasgupta, K. Tsuno, B. Monteleone, N. Shimizu, Carbon and sulfur budget of the silicate Earth explained by accretion of differentiated planetary embryos. *Nat. Geosci.* **9**, 781–785 (2016).

12. Y. Li, M. Wiedenbeck, B. Monteleone, R. Dasgupta, G. Costin, Z. Gao, W. Lu, Nitrogen and carbon fractionation in planetary magma oceans and origin of the superchondritic C/N ratio in the bulk silicate Earth. *Earth Planet. Sci. Lett.* **605**, 118032 (2023).

13. L. Piani, Y. Marrocchi, T. Rigaudier, L. G. Vacher, D. Thomassin, B. Marty, Earth's water may have been inherited from material similar to enstatite chondrite meteorites. *Science* **369**, 1110–1113 (2020).

14. K. J. Walsh, A. Morbidelli, S. N. Raymond, D. P. O'Brien, A. M. Mandell, A low mass for Mars from Jupiter's early gas-driven migration. *Nature* **475**, 206–209 (2011).

15. A. Izidoro, R. Dasgupta, S. N. Raymond, R. Deienno, B. Bitsch, A. Isella, Planetesimal rings as the cause of the Solar System's planetary architecture. *Nat. Astron.* **6**, 357–366 (2021).

16. G. Budde, C. Burkhardt, G. A. Brennecka, M. Fischer-Gödde, T. S. Kruijer, T. Kleine, Molybdenum isotopic evidence for the origin of chondrules and a distinct genetic heritage of carbonaceous and non-carbonaceous meteorites. *Earth Planet. Sci. Lett.* **454**, 293–303 (2016).

17. E. R. D. Scott, "Iron meteorites: Composition, age, and origin," in *Oxford Research Encyclopedia of Planetary Science* (Oxford Academic, New York, 2020).

18. D. S. Grewal, R. Dasgupta, A. K. Holmes, G. Costin, Y. Li, K. Tsuno, The fate of nitrogen during core-mantle separation on Earth. *Geochim. Cosmochim. Acta* **251**, 87–115 (2019).

19. T. A. Suer, J. Siebert, L. Remusat, N. Menguy, G. Fiquet, A sulfur-poor terrestrial core inferred from metal–silicate partitioning experiments. *Earth Planet. Sci. Lett.* **469**, 84–97 (2017).

20. L. Shi, W. Lu, T. Kagoshima, Y. Sano, Z. Gao, Z. Du, Y. Liu, Y. Fei, Y. Li, Nitrogen isotope evidence for Earth's heterogeneous accretion of volatiles. *Nat. Commun.* **13**, 1–15 (2022).

21. D. Pathak, R. Dasgupta, Nitrogen inventory of iron meteorite parent bodies constrained by nitrogen partitioning between Fe-rich solid and liquid alloys. *Geochim. Cosmochim. Acta* **371**, 199–213 (2024).

22. D. S. Grewal, P. D. Asimow, Origin of the superchondritic carbon/nitrogen ratio of the bulk silicate Earth—An outlook from iron meteorites. *Geochim. Cosmochim. Acta* **344**, 146–159 (2023).

23. M. M. Hirschmann, E. A. Bergin, G. A. Blake, F. J. Ciesla, J. Li, Early volatile depletion on planetesimals inferred from C–S systematics of iron meteorite parent bodies. *Proc. Natl. Acad. Sci. U.S.A.* **118**, e2026779118 (2021).

24. D. S. Grewal, S. Bhattacharjee, G. D. Mardaru, P. D. Asimow, Tracing the origin of volatiles on Earth using nitrogen isotope ratios in iron meteorites. *Geochim. Cosmochim. Acta* **388**, 34–47 (2025).

25. K. Lodders, Solar system abundances and condensation temperatures of the elements. *Astrophys. J.* **591**, 1220–1247 (2003).

26. M. Roskosz, M. A. Bouhifd, A. P. Jephcoat, B. Marty, B. O. Mysen, Nitrogen solubility in molten metal and silicate at high pressure and temperature. *Geochim. Cosmochim. Acta* **121**, 15–28 (2013).

27. K. Righter, K. M. Pando, L. Danielson, C. T. Lee, Partitioning of Mo, P and other siderophile elements (Cu, Ga, Sn, Ni, Co, Cr, Mn, V, and W) between metal and silicate melt as a function of temperature and silicate melt composition. *Earth Planet. Sci. Lett.* **291**, 1–9 (2010).

28. J. Siebert, A. Corgne, F. J. Ryerson, Systematics of metal–silicate partitioning for many siderophile elements applied to Earth's core formation. *Geochim. Cosmochim. Acta* **75**, 1451–1489 (2011).

29. N. Ikuta, N. Sakamoto, S. Tagawa, K. Hirose, Y. Tsutsumi, S. Yokoo, H. Yurimoto, Pressure dependence of metal–silicate partitioning explains the mantle phosphorus abundance. *Sci. Rep.* **14**, 1–8 (2024).

30. C. R. Walton, S. Ewens, J. D. Coates, R. E. Blake, N. J. Planavsky, C. Reinhard, P. Ju, J. Hao, M. A. Pasek, Phosphorus availability on the early Earth and the impacts of life. *Nat. Geosci.* **16**, 399–409 (2023).

31. B. Marty, The origins and concentrations of water, carbon, nitrogen and noble gases on Earth. *Earth Planet. Sci. Lett.* **313-314**, 56–66 (2012).

32. B. Zhang, N. L. Chabot, A. E. Rubin, Compositions of carbonaceous-type asteroidal cores in the early solar system. *Sci. Adv.* **8**, 5781 (2022).

33. B. Zhang, N. L. Chabot, A. E. Rubin, Compositions of iron-meteorite parent bodies constrain the structure of the protoplanetary disk. *Proc. Natl. Acad. Sci. U.S.A.* **121**, e2306995121 (2024).

34. C. M. Corrigan, N. L. Chabot, T. J. McCoy, W. F. McDonough, H. C. Watson, S. A. Saslow, R. D. Ash, The iron–nickel–phosphorus system: Effects on the distribution of trace elements during the evolution of iron meteorites. *Geochim. Cosmochim. Acta* **73**, 2674–2691 (2009).

35. I. A. Franchi, I. P. Wright, C. T. Pillinger, Constraints on the formation conditions of iron meteorites based on concentrations and isotopic compositions of nitrogen. *Geochim. Cosmochim. Acta* **57**, 3105–3121 (1993).

36. C. A. Prombo, R. N. Clayton, Nitrogen isotopic compositions of iron meteorites. *Geochim. Cosmochim. Acta* **57**, 3749–3761 (1993).

37. S. V. S. Murty, K. Marti, Nitrogen isotopic signatures in Cape York: Implications for formation of Group III A irons. *Geochim. Cosmochim. Acta* **58**, 1841–1848 (1994).

38. P. N. Shukla, P. S. Goel, Total nitrogen in iron meteorites. *Earth Planet. Sci. Lett.* **52**, 251–258 (1981).

39. S. V. S. Murty, P. S. Goel, D. V. Minh, Y. A. Shukolyukov, Nitrogen and xenon in acid residues of iron meteorites. *Geochim. Cosmochim. Acta* **47**, 1061–1068 (1983).

40. K. Zhai, Y. Yin, S. Zhai, New constraints on the abundances of phosphorus and sulfur in the lunar core: High-pressure and high-temperature experimental study of the FeSP ternary system. *Geochim. Cosmochim. Acta* **334**, 1–13 (2022).

41. Y. Yin, Z. Li, S. Zhai, The phase diagram of the Fe-P binary system at 3 GPa and implications for phosphorus in the lunar core. *Geochim. Cosmochim. Acta* **254**, 54–66 (2019).

42. A. J. Stewart, M. W. Schmidt, Sulfur and phosphorus in the Earth's core: The Fe-P-S system at 23 GPa. *Geophys. Res. Lett.* **34**, L13201 (2007).

43. A. J. Stewart, W. van Westrenen, M. W. Schmidt, D. Günther, Minor element partitioning between fcc Fe metal and Fe–S liquid at high pressure: The role of crystal lattice strain. *Earth Planet. Sci. Lett.* **284**, 302–309 (2009).

44. D. Huang, J. Siebert, P. Sossi, E. Kubik, G. Avice, M. Murakami, Nitrogen sequestration in the core at megabar pressure and implications for terrestrial accretion. *Geochim. Cosmochim. Acta* **376**, 100–112 (2024).

45. D. S. Grewal, N. X. Nie, B. Zhang, A. Izidoro, P. D. Asimow, Accretion of the earliest inner Solar System planetesimals beyond the water snowline. *Nat. Astron.* **8**, 290–297 (2024).

46. C. D. Hilton, R. D. Ash, R. J. Walker, Chemical characteristics of iron meteorite parent bodies. *Geochim. Cosmochim. Acta* **318**, 112–125 (2022).

47. D. S. Grewal, J. D. Seales, R. Dasgupta, Internal or external magma oceans in the earliest protoplanets—Perspectives from nitrogen and carbon fractionation. *Earth Planet. Sci. Lett.* **598**, 117847 (2022).

48. C. M. O. D. Alexander, G. D. Cody, B. T. De Gregorio, L. R. Nittler, R. M. Stroud, The nature, origin and modification of insoluble organic matter in chondrites, the major source of Earth's C and N. *Geochemistry* **77**, 227–256 (2017).

49. V. K. Pearson, M. A. Sephton, I. A. Franchi, J. M. Gibson, I. Gilmour, Carbon and nitrogen in carbonaceous chondrites: Elemental abundances and stable isotopic compositions. *Meteorit. Planet. Sci.* **41**, 1899–1918 (2006).

50. G. D. Cody, C. M. O. D. Alexander, H. Yabuta, A. L. D. Kilcoyne, T. Araki, H. Ade, P. Dera, M. Fogel, B. Militzer, B. O. Mysen, Organic thermometry for chondritic parent bodies. *Earth Planet. Sci. Lett.* **272**, 446–455 (2008).

51. M. A. Pasek, Phosphorus volatility in the early solar nebula. *Icarus* **317**, 59–65 (2019).

52. N. Dzyurkevich, M. Flock, N. J. Turner, H. Klahr, T. Henning, Trapping solids at the inner edge of the dead zone: 3-D global MHD simulations. *Astron. Astrophys.* **515**, A70 (2010).

53. M. R. Jankovic, J. E. Owen, S. Mohanty, J. C. Tan, MRI-active inner regions of protoplanetary discs. I. A detailed model of disc structure. *Mon. Not. R. Astron. Soc.* **504**, 280–299 (2021).

54. G. Wurm, H. Haack, Outward transport of CAIs during FU-Orionis events. *Meteorit. Planet. Sci.* **44**, 689–699 (2009).

55. A. P. Boss, Mixing and transport of isotopic heterogeneity in the early solar system. *Annu. Rev. Earth Planet. Sci.* **40**, 23–43 (2012).

56. F. J. Ciesla, Outward transport of high-temperature materials around the midplane of the solar nebula. *Science* **318**, 613–615 (2007).

57. J. Y. Hu, F. L. H. Tissot, R. T. C. Marquez, O. Shorttle, C. J. Clarke, A. D. Sellek, N. Dauphas, B. L. A. Charlier, I. Leya, R. Yokochi, T. J. Ireland, H. M. Williams, Rare earth element nucleosynthetic anomalies and dust transport in the protoplanetary disk. *Sci. Adv.* **11**, 3148 (2025).

58. D. C. Hezel, S. S. Russell, A. J. Ross, A. T. Kearsley, Modal abundances of CAIs: Implications for bulk chondrite element abundances and fractionations. *Meteorit. Planet. Sci.* **43**, 1879–1894 (2008).

59. M. M. Grady, I. P. Wright, L. P. Carr, C. T. Pillinger, Compositional differences in enstatite chondrites based on carbon and nitrogen stable isotope measurements. *Geochim. Cosmochim. Acta* **50**, 2799–2813 (1986).

60. A. Morbidelli, G. Libourel, H. Palme, S. A. Jacobson, D. C. Rubie, Subsolar Al/Si and Mg/Si ratios of non-carbonaceous chondrites reveal planetesimal formation during early condensation in the protoplanetary disk. *Earth Planet. Sci. Lett.* **538**, 116220 (2020).

61. A. Morbidelli, K. Baillié, K. Batygin, S. Charnoz, T. Guillot, D. C. Rubie, T. Kleine, Contemporary formation of early Solar System planetesimals at two distinct radial locations. *Nat. Astron.* **6**, 72–79 (2021).

62. A. Izidoro, B. Bitsch, R. Dasgupta, The effect of a strong pressure bump in the Sun's natal disk: Terrestrial planet formation via planetesimal accretion rather than pebble accretion. *Astrophys. J.* **915**, 62 (2021).

63. N. Dauphas, The isotopic nature of the Earth's accreting material through time. *Nature* **541**, 521–524 (2017).

64. N. Dauphas, T. Hopp, D. Nesvorný, Bayesian inference on the isotopic building blocks of Mars and Earth. *Icarus* **408**, 115805 (2024).

65. W. F. McDonough, Compositional model for the Earth's core. *Treatise Geochem.* **2–9**, 547–568 (2003).

66. H. Palme, H. O'Neill, Cosmochemical estimates of mantle composition. *Treatise Geochem.* **3**, 1–39 (2014).

67. C. Burkhardt, F. Spitzer, A. Morbidelli, G. Budde, J. H. Render, T. S. Kruijer, T. Kleine, Terrestrial planet formation from lost inner solar system material. *Sci. Adv.* **7**, 7601 (2021).

68. C. B. Moore, E. K. Gibson, Nitrogen abundances in chondritic meteorites. *Science* **163**, 174–176 (1969).

69. M. Landeau, R. Deguen, D. Phillips, J. A. Neufeld, V. Lherm, S. B. Dalziel, Metal-silicate mixing by large Earth-forming impacts. *Earth Planet. Sci. Lett.* **564**, 116888 (2021).

70. J. D. Kendall, H. J. Melosh, Differentiated planetesimal impacts into a terrestrial magma ocean: Fate of the iron core. *Earth Planet. Sci. Lett.* **448**, 24–33 (2016).

71. R. Deguen, P. Olson, P. Cardin, Experiments on turbulent metal-silicate mixing in a magma ocean. *Earth Planet. Sci. Lett.* **310**, 303–313 (2011).

72. D. C. Rubie, H. J. Melosh, J. E. Reid, C. Liebske, K. Righter, Mechanisms of metal–silicate equilibration in the terrestrial magma ocean. *Earth Planet. Sci. Lett.* **205**, 239–255 (2003).

73. K. Righter, Prediction of metal-silicate partition coefficients for siderophile elements: An update and assessment of PT conditions for metal-silicate equilibrium during accretion of the Earth. *Earth Planet. Sci. Lett.* **304**, 158–167 (2011).

74. P. A. Sossi, D. J. Bower, Homogeneous accretion of the Earth in the inner Solar System. *Nat. Astron.* **2026**, 1–8 (2026).

75. D. Pathak, R. Dasgupta, The existing frameworks of delivery of major volatiles and the feasibility of Mars-mass planetary embryos as the major volatile contributors to bulk silicate Earth. *Astrobiology*, 15311074251365197 (2025).

76. M. M. Hirschmann, Constraints on the early delivery and fractionation of Earth's major volatiles from C/H, C/N, and C/S ratios. *Am. Mineral.* **101**, 540–553 (2016).

77. F. Gaillard, F. Bernadou, M. Roskosz, M. A. Bouhifd, Y. Marrocchi, G. Iacono-Marziano, M. Moreira, B. Scaillet, G. Rogerie, Redox controls during magma ocean degassing. *Earth Planet. Sci. Lett.* **577**, 117255 (2022).

78. J. T. Gu, B. Peng, X. Ji, J. Zhang, H. Yang, S. Hoyos, M. M. Hirschmann, E. S. Kite, R. A. Fischer, Composition of Earth's initial atmosphere and fate of accreted volatiles set by core formation and magma ocean redox evolution. *Earth Planet. Sci. Lett.* **629**, 118618 (2024).

79. T. Yoshioka, D. Nakashima, T. Nakamura, S. Shcheka, H. Keppler, Carbon solubility in silicate melts in equilibrium with a CO-CO_2 gas phase and graphite. *Geochim. Cosmochim. Acta* **259**, 129–143 (2019).

80. H. Sakuraba, H. Kurokawa, H. Genda, K. Ohta, Numerous chondritic impactors and oxidized magma ocean set Earth's volatile depletion. *Sci. Rep.* **11**, 1–14 (2021).

81. P. Cartigny, B. Marty, Nitrogen isotopes and mantle geodynamics: The emergence of life and the atmosphere–crust–mantle connection. *Elements* **9**, 359–366 (2013).

82. K. Tsuno, R. Dasgupta, Melting phase relation of nominally anhydrous, carbonated pelitic-eclogite at 2.5-3.0 GPa and deep cycling of sedimentary carbon. *Contrib. Mineral. Petrol.* **161**, 743–763 (2011).

83. M. Vogt, M. Trieloff, U. Ott, J. Hopp, W. H. Schwarz, Solar noble gases in an iron meteorite indicate terrestrial mantle signatures derive from Earth's core. *Commun. Earth Environ.* **2**, 1–7 (2021).

84. N. L. Chabot, A. J. Campbell, W. F. McDonough, D. S. Draper, C. B. Agee, M. Humayun, H. C. Watson, E. Cottrell, S. A. Saslow, The Fe–C system at 5 GPa and implications for Earth's core. *Geochim. Cosmochim. Acta* **72**, 4146–4158 (2008).

85. N. L. Chabot, E. A. Wollack, W. F. Mcdonough, R. D. Ash, S. A. Saslow, Experimental determination of partitioning in the Fe-Ni system for applications to modeling meteoritic metals. *Meteorit. Planet. Sci.* **52**, 1133–1145 (2017).

86. E. Kaminski, A. Limare, B. Kenda, M. Chaussidon, Early accretion of planetesimals unraveled by the thermal evolution of the parent bodies of magmatic iron meteorites. *Earth Planet. Sci. Lett.* **548**, 116469 (2020).

87. J. Yang, J. I. Goldstein, Metallographic cooling rates of the IIIAB iron meteorites. *Geochim. Cosmochim. Acta* **70**, 3197–3215 (2006).

88. J. T. Wasson, Y. Matsunami, A. E. Rubin, Silica and pyroxene in IVA irons; possible formation of the IVA magma by impact melting and reduction of L-LL-chondrite materials followed by crystallization and cooling. *Geochim. Cosmochim. Acta* **70**, 3149–3172 (2006).

89. J. Yang, J. I. Goldstein, E. R. D. Scott, Metallographic cooling rates and origin of IVA iron meteorites. *Geochim. Cosmochim. Acta* **72**, 3043–3061 (2008).

90. J. Yang, J. I. Goldstein, J. R. Michael, P. G. Kotula, E. R. D. Scott, Thermal history and origin of the IVB iron meteorites and their parent body. *Geochim. Cosmochim. Acta* **74**, 4493–4506 (2010).

91. D. Andrault, N. Bolfan-Casanova, G. Lo Nigro, M. A. Bouhifd, G. Garbarino, M. Mezouar, Solidus and liquidus profiles of chondritic mantle: Implication for melting of the Earth across its history. *Earth Planet. Sci. Lett.* **304**, 251–259 (2011).

92. K. Litasov, E. Ohtani, Phase relations and melt compositions in CMAS–pyrolite–H_2O system up to 25 GPa. *Phys. Earth Planet. In.* **134**, 105–127 (2002).

93. A. Boujibar, D. Andrault, M. A. Bouhifd, N. Bolfan-Casanova, J. L. Devidal, N. Trcera, Metal–silicate partitioning of sulphur, new experimental and thermodynamic constraints on planetary accretion. *Earth Planet. Sci. Lett.* **391**, 42–54 (2014).

94. C. R. M. Jackson, E. Cottrell, Z. Du, N. R. Bennett, Y. Fei, High pressure redistribution of nitrogen and sulfur during planetary stratification. *Geochem. Persp. Lett.* **18**, 37–42 (2021).

95. E. Mallick, K. Prissel, K. Righter, C. R. M. Jackson, The fate of nitrogen in deep magma oceans. *Geochim. Cosmochim. Acta* **394**, 298–318 (2025).

96. J. T. Wasson, G. W. Kallemeyn, Compositions of chondrites. *Philos. Trans. A. Math. Phys. Sci.* **325**, 535–544 (1988).

97. B. Marty, M. Almayrac, P. H. Barry, D. V. Bekaert, M. W. Broadley, D. J. Byrne, C. J. Ballentine, A. Caracausi, An evaluation of the C/N ratio of the mantle from natural CO_2-rich gas analysis: Geochemical and cosmochemical implications. *Earth Planet. Sci. Lett.* **551**, 116574 (2020).

98. E. K. Gibson, C. B. Moore, The distribution of total nitrogen in iron meteorites. *Geochim. Cosmochim. Acta* **35**, 877–890 (1971).

99. B. K. Kothari, P. S. Goel, Total nitrogen in meteorites. *Geochim. Cosmochim. Acta* **38**, 1493–1507 (1974).

100. N. Sugiura, Y. Ikeda, S. Zashu, J. T. Wasson, Nitrogen-isotopic compositions of IIIE iron meteorites. *Meteorit. Planet. Sci.* **35**, 749–756 (2000).

101. R. O. Pepin, R. H. Becker, R. O. Pepin, R. H. Becker, Nitrogen isotopes in iron meteorites. *Metic* **17**, 269 (1982).

102. H. A. Tornabene, C. D. Hilton, K. R. Bermingham, R. D. Ash, R. J. Walker, Genetics, age and crystallization history of group IIC iron meteorites. *Geochim. Cosmochim. Acta* **288**, 36–50 (2020).

103. J. T. Wasson, H. Huber, Compositional trends among IID irons; their possible formation from the P-rich lower magma in a two-layer core. *Geochim. Cosmochim. Acta* **70**, 6153–6167 (2006).

104. H. A. Tornabene, R. D. Ash, R. J. Walker, K. R. Bermingham, Genetics, age, and crystallization history of group IC iron meteorites. *Geochim. Cosmochim. Acta* **340**, 108–119 (2023).

105. J. T. Wasson, H. Huber, D. J. Malvin, Formation of IIAB iron meteorites. *Geochim. Cosmochim. Acta* **71**, 760–781 (2007).

106. N. Chabot, B. Zhang, A revised trapped melt model for iron meteorites applied to the IIIAB group. *Meteorit. Planet. Sci.* **57**, 200–227 (2021).

107. A. E. Rubin, B. Zhang, N. L. Chabot, IVA iron meteorites as late-stage crystallization products affected by multiple collisional events. *Geochim. Cosmochim. Acta* **331**, 1–17 (2022).

108. E. M. Chiappe, R. D. Ash, R. J. Walker, Age, genetics, and crystallization sequence of the group IIIE iron meteorites. *Geochim. Cosmochim. Acta* **354**, 51–61 (2023).
